# Supplementary material for: SMARCAD1 ATPase activity is required to silence endogenous retroviruses in embryonic stem cells
Source: Nat Commun. 2019 Mar 22;10:1335. doi: 10.1038/s41467-019-09078-0 (PMC6430823; doi:10.1038/s41467-019-09078-0)
Supplement: Supplementary file 1 — Supplementary Information [file 41467_2019_9078_MOESM1_ESM.pdf]

Supplementary Information

SMARCAD1 ATPase activity is required to silence endogenous retroviruses in embryonic stem cells

Parysatis Sachs, Dong Ding, Philipp Bergmaier, Boris Lamp, Christina Schlagheck, Florian Finkernagel, Andrea Nist, Thorsten Stiewe, Jacqueline E. Mermoud

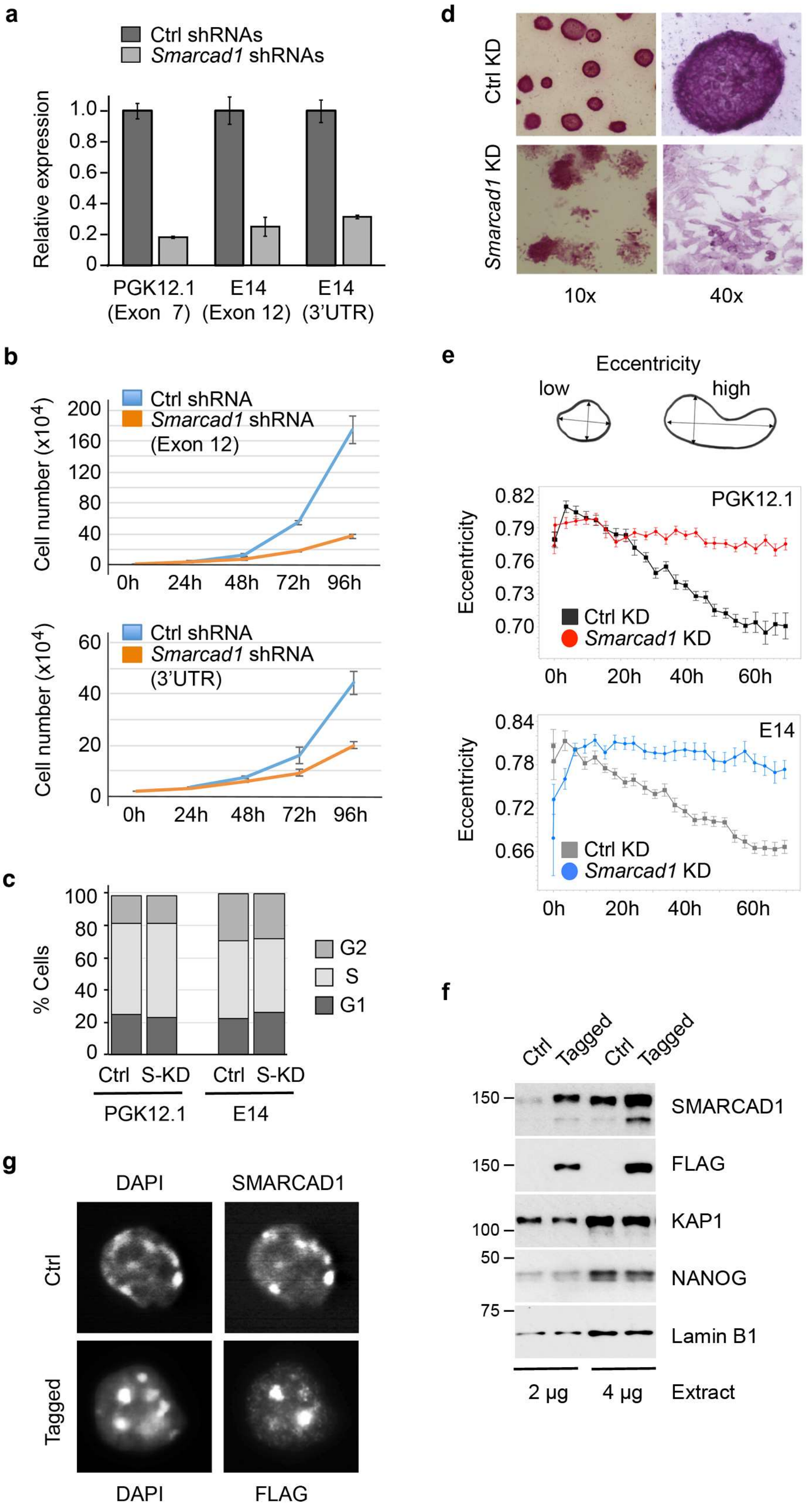

Supplementary Figure 1
Sachs et al.

Supplementary Figure 1. Related to Figure 1. Characterization of mESCs either stably depleted for SMARCAD1 or expressing triple FLAG tagged SMARCAD1.

(a-e) Stable knockdown (KD) of *Smarcad1* in female (PGK12.1) and male (E14) ESCs with small hairpin RNAs (shRNAs) reduces *Smarcad1* levels and results in changes in cell morphology and growth.

(a) RT-qPCR analyses show *Smarcad1* mRNA levels after depletion through shRNAs (left to right: targeting Exon 7, Exon 12 and the 3'UTR of *Smarcad1* respectively) compared to unspecific shRNAs (Ctrl). Error bars indicate mean ± S.D. of technical triplicates.

(b) Growth curves of E14 ESCs depleted for SMARCAD1 show reduced proliferation compared to control knockdowns. Triplicate cultures were counted at 24 hour intervals and error bars show mean ± S.D.

(c) Cell cycle distribution of *Smarcad1* and Ctrl KD cells determined by flow cytometry. Left, PGK12.1 ESCs treated with an shRNA targeting Exon 7 of *Smarcad1*. Right, E14 ESCs treated with an shRNA targeting the 3'UTR of *Smarcad1*. The percentage of cells in G1/S/G2 is shown.

(d) Alkaline phosphatase staining reveals that stable knockdown of *Smarcad1* in PGK12.1 cells with an shRNA targeting exon 7 results in the loss of their typical morphology and their strong alkaline phosphatase staining (violet staining). The used magnification is shown.

(e) *Smarcad1* knockdown cells are less spherical than control knockdown cells. PGK12.1 ESCs stably expressing an shRNA targeting Exon 7 and E14 ESCs stably expressing an shRNA targeting the 3'UTR of *Smarcad1* and appropriate control knockdown cells were plated at six different concentrations ranging from 10 K to 312 cells/ 96 well (n=3). Their average eccentricity was analysed in real time with an IncuCyte in three hour intervals for three days. A representative example of this analysis is shown, the error bars depicting the S.D. from triplicates.

(f and g) Tagged SMARCAD1 protein expression in stably transfected PGK12.1 ESCs.

(f) Expression level of 3×FLAG-tagged SMARCAD1 compared to endogenous protein levels from a control cell line transfected with the FLAG empty vector (Ctrl). Immunoblots on 2 or 4 µg total extracts were probed with a SMARCAD1 antibody, detecting both endogenous and tagged SMARCAD1, and a FLAG antibody, detecting only the tagged version of SMARCAD1. KAP1 and NANOG levels remain unchanged upon overexpression of SMARCAD1 when compared to the control. Lamin B1 serves as a loading control.

(g) Immunofluorescence analysis of 3×FLAG tagged-SMARCAD1 expressing PGK12.1 ESCs compared to cells transfected with the control plasmid. Soluble proteins were extracted before fixing and nuclei were counterstained with DAPI. DAPI-dense areas represent pericentric heterochromatin. Endogenous SMARCAD1 (top panel, Ctrl cell line stained with SMARCAD1 antibody) displays nuclear localisation and enrichment at pericentric heterochromatin in 50% of the cell population (n>120). The localization of the tagged SMARCAD1 protein (lower panel, FLAG expressing ESCs stained with FLAG antibody) is comparable with the nuclear localisation of the endogenous protein; with 37% of cells (n>120) showing enrichment of FLAG-SMARCAD1 at pericentric heterochromatin.

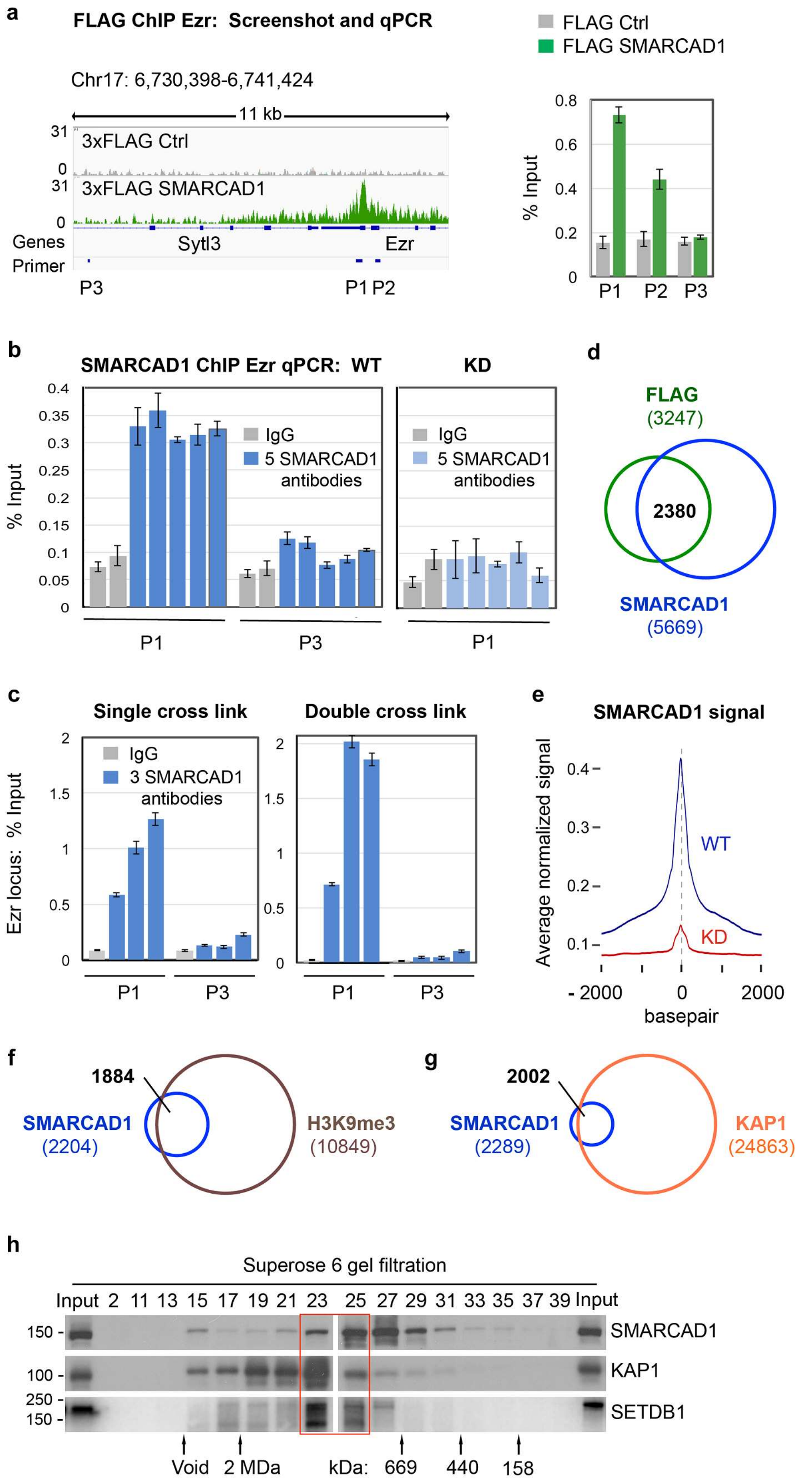

Supplementary Figure 2
Sachs et al.

Supplementary Figure 2. Related to Figure 1. Validation of FLAG ChIP-seq and optimization of endogenous SMARCAD1 ChIP.

(a) Validation of FLAG-SMARCAD1 binding in ESCs. Left, genome browser screenshot of FLAG ChIP-seq experiments examining tagged SMARCAD1 occupancy in control (3×FLAG-empty vector) or 3×FLAG-SMARCAD1 expressing PGK12.1 ESCs over a sample locus (*Ezr*). The genomic coordinates of chromosome 17 are indicated. The location of ChIP-qPCR primer pairs (P1-P3) is shown: P1 pair lies within the called peak, P2 pair 1.5 kb downstream and P3 pair 7.8 kb upstream. Right, chromatin immunoprecipitations using an antibody against FLAG followed by qPCR were performed to determine the distribution of FLAG-SMARCAD1 across this region of chromosome 17. FLAG-SMARCAD1 was detected at the 3' end of the *Ezr* gene (P1, P2), but was absent from the upstream regions (P3).

(b) ChIP-qPCR analysis of endogenous SMARCAD1 at candidate loci identified by FLAG ChIP-seq of tagged SMARCAD1. Depicted here is the binding of endogenous SMARCAD1 in single cross linked PGK12.1 cells on the locus *Ezr* characterized in (a). The primers are as in (a). This binding is specific as it is abolished in cells lacking SMARCAD1 (KD, right panel) or with primers that amplify a region of the genome 7.8 kb upstream of *Ezr* (P3). A number of different SMARCAD1 specific antibodies were used, from left to right: A301-592A, A301-593A, Anti-CUE, ab67548, PAB15737 (and not shown here HPA016737). These antibodies are described in Supplementary Table 2.

(c) Comparison of ChIP efficiency of endogenous SMARCAD1 proteins in Single (FA) or Double crosslinked (DSG+FA) E14 ESC chromatin. Applying two sequential fixation steps (DSG+FA) achieved an improved enrichment over the *Ezr* locus, but did not lead to a general increase in precipitated material as illustrated with primer pair P3 described in (a). Antibodies from left to right: A301-592A, PAB15737 and HPA016737. This improvement was especially apparent for the SMARCAD1 antibody PAB15737 which was used in subsequent ChIP-seq experiments. ChIP-qPCR results in (a, b and c) represent the mean ± S.E. of technical triplicates.

(d) Venn diagramm showing the overlap of ChIP-seq peaks identified upon FLAG ChIP in FLAG-SMARCAD1 expressing PGK12.1 ESCs (green) and SMARCAD1 ChIP in wild-type PGK12.1 ESCs (blue). Numbers in parentheses indicate numbers of filtered peaks obtained with antibodies against endogenous (5669) and FLAG tagged (3247) SMARCAD1. The intersection, 2380 peaks, are considered high confidence binding sites.

(e) Averaged normalized signal over the 2380 SMARCAD1 binding sites identified by ChIP-seq for endogenous SMARCAD1 in PGK12.1 (blue) and SMARCAD1 knockdown (KD, red) ESCs. Dashed line indicates peak summit. The x axis depicts the distance from the peak summit (bp) and the y axis the average, normalized signal (tag counts per million mapped reads).

(f, g) Venn diagrams revealing a striking overlap of high confidence SMARCAD1 targets, namely the intersection of SMARCAD1 and FLAG-SMARCAD1 peaks (this study), with the H3K9me3 mark (this study) and with KAP1 targets [1].

(f) Out of 2204 SMARCAD1 intersection peaks, 1884 overlapped with H3K9me3.

(g) In total, 2002 peaks of the SMARCAD1 intersection (2289) overlapped with the KAP1 dataset published [1].

(**h**) Gel filtration analysis of SMARCAD1, KAP1 and SETDB1 in ESCs**.** Nuclear extract was size fractionated on a Superose 6 gel filtration column. Fractions were analysed by SDS

PAGE and probed with the indicated antibodies. Fraction numbers are indicated at the top. Molecular weights of size standards are indicated at the bottom. Input loading was 2 % (SETDB1) and 4% (SMARCAD1, KAP1) of extract.

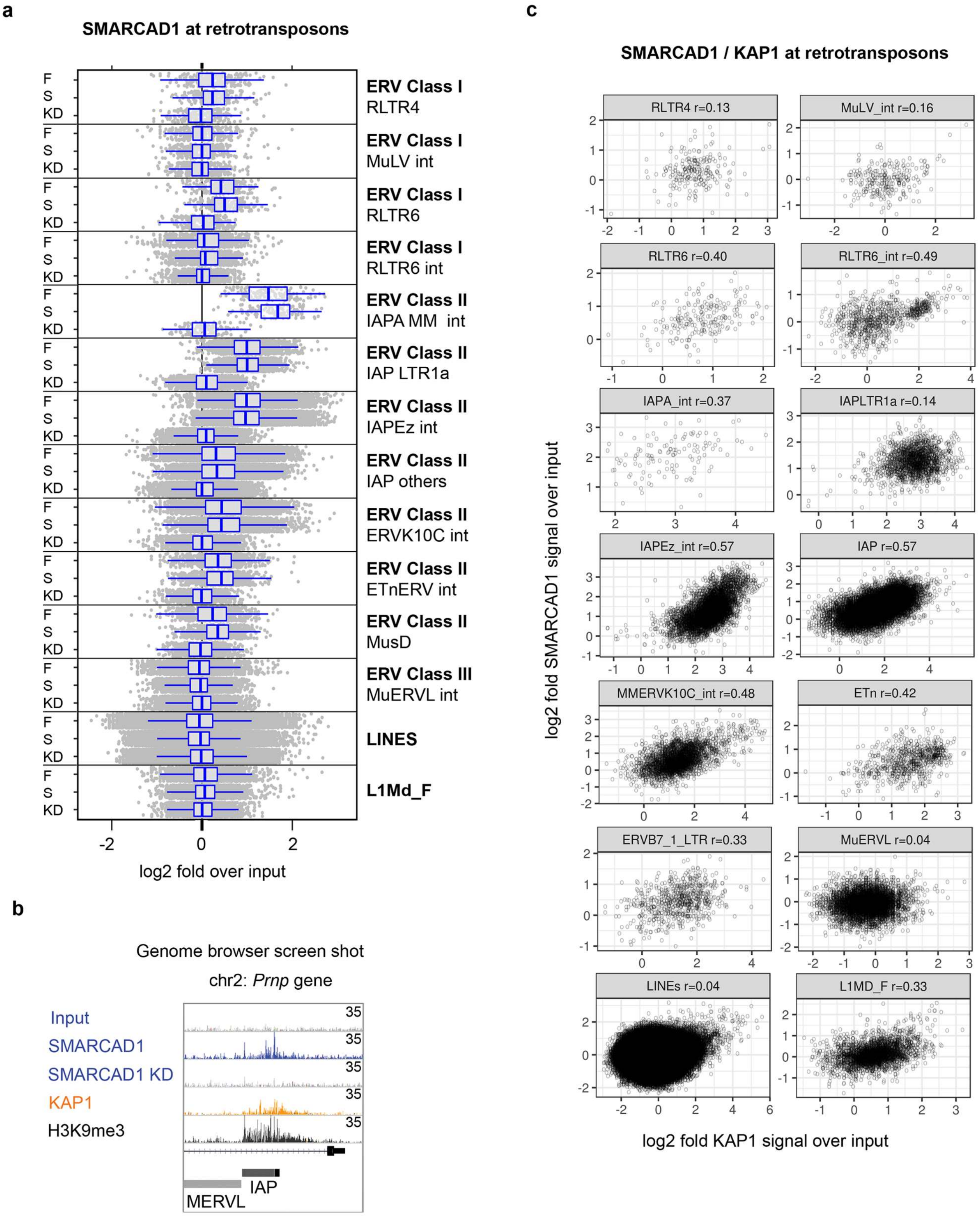

Supplementary Figure 3
Sachs et al.

Supplementary Figure 3. Related to Figure 2. FLAG-SMARCAD1, endogenous SMARCAD1 and KAP1 are enriched at specific sub-classes of retrotransposons in mESCs.

(a) Combined box- and jitterplot depicting the log2 fold ratio over input of TPM in FLAG-SMARCAD1 (F) and endogenous SMARCAD1 (S) ChIP-Seq experiments in PGK12.1 ESCs; KD, endogenous SMARCAD1 ChIP in *Smarcad1* knockdown PGK12.1 ESCs. Each dot represents a single repetitive element. In the boxplot centre lines shows the median; lower and upper box lines correspond to 25th and 75th percentiles. Names of repeat subfamilies were taken from the UCSC supplied RepeatMasker information: “IAP others” refers to all repeat elements named IAP except the ones shown separately (IAPA_MM-int, IAPEz-int and IAPLTR1a); MusD (ERV7 1 LTR - ERVB7 1 LTR); MuERVL int combines MERVL_2A-int and MERVL-int elements; LINEs refers to all repetitive elements of the repeat family LINE, except the ones shown separately (all elements that have L1Md_F in their name).

(b) Representative genomic region illustrating co-localisation of SMARCAD1 with KAP1 and H3K9me3 at ERV class II elements (IAPEz) but no accumulation at class III elements (MERVL). Tracks for SMARCAD1 ChIP-seq, input and KD control, and for H3K9me3 ChIP-seq in PGK12.1 ESCs are shown. KAP1 ChIP-seq tracks are from [1]. Numbers on the right indicate *y* axis scale. Repeat data were retrieved from the RepeatMasker using the UCSC table browser.

(c) Comparison of SMARCAD1 and KAP1 binding on retrotransposons. Individual members of the families described in Figure 2a are shown. Correlations were calculated using Spearman's correlation.

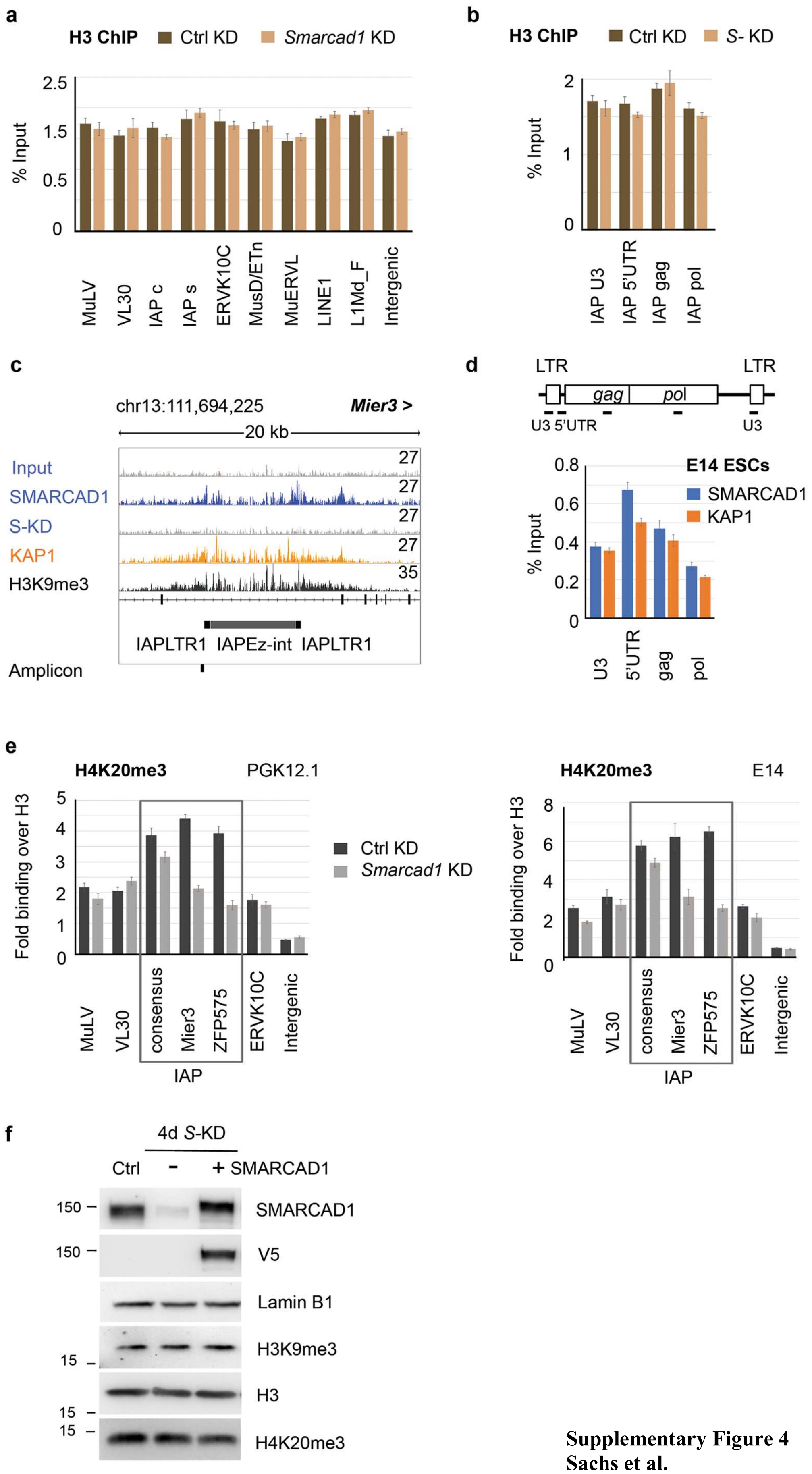

Supplementary Figure 4
Sachs et al.

Supplementary Figure 4. Related to Figure 3. In mESCs, SMARCAD1 binds specific LTR retrotransposons, preserving H3K9me3 and H4K20me3 levels at IAPs.

(a and b) H3 occupancy at retrotransposons is not altered in *Smarcad1* knockdown ESCs. Chromatin analysed corresponds to samples described in Figures 3a and 3b. ChIP-qPCR results are the mean ± S.E. of technical triplicates.

(c) A genome browser screenshot of the *Mier3* genomic locus on chromosome 13 which harbours an IAP element. The arrowhead indicates the direction of gene transcription. ChIP-seq signals for endogenous SMARCAD1, with input and knockdown (KD) control read densities, H3K9me3 (this study) and KAP1 [1] are shown, along with the locations of IAP elements and the primer amplicon designed in this study.

(d) Top, IAP structure like in Figure 3b. Below, SMARCAD1 and KAP1 ChIP-qPCR in E14 ESCs across IAP elements. SMARCAD1 binding at the 5'UTR of IAP elements is more pronounced than at the coding regions in both PGK12.1 ESCs (Figure 3b) and E14 ESCs (shown here). A representative example from three biological replicates is shown; error bars denote mean ± S.E.

(e) SMARCAD1 depleted cells show a reduction in H4K20me3 levels at selected ERVs, especially at IAP elements (boxed). ChIP-qPCR analysis in stable (left panel; PGK12.1) and four day (right panel; E14) *Smarcad1* knockdown ESCs. Real-time qPCR was carried out in triplicates and enrichment (mean ± S.E.) is presented as the fold change of H4K20me3 over percent input of H3.

(f) *Smarcad1* knockdown and reconstitution with a *Smarcad1* transgene does not affect total H3K9me3 or H4K20me3 levels. Western blot analysis of E14 ESCs either untreated (Ctrl) or depleted for SMARCAD1 (*S*-KD) for four days (compare lanes 1 and 2), when these cells were used for ChIP-qPCR analysis of H3, H3K9me3 and H4K20me3. Lane 3 shows the expression of wild-type, tagged SMARCAD1 in *Smarcad1* knockdown cells. The anti-SMARCAD1 antibody recognizes both endogenous and exogenous SMARCAD1, the anti-V5 antibody recognizes only exogenous SMARCAD1. Lamin B1 and H3 served as loading controls.

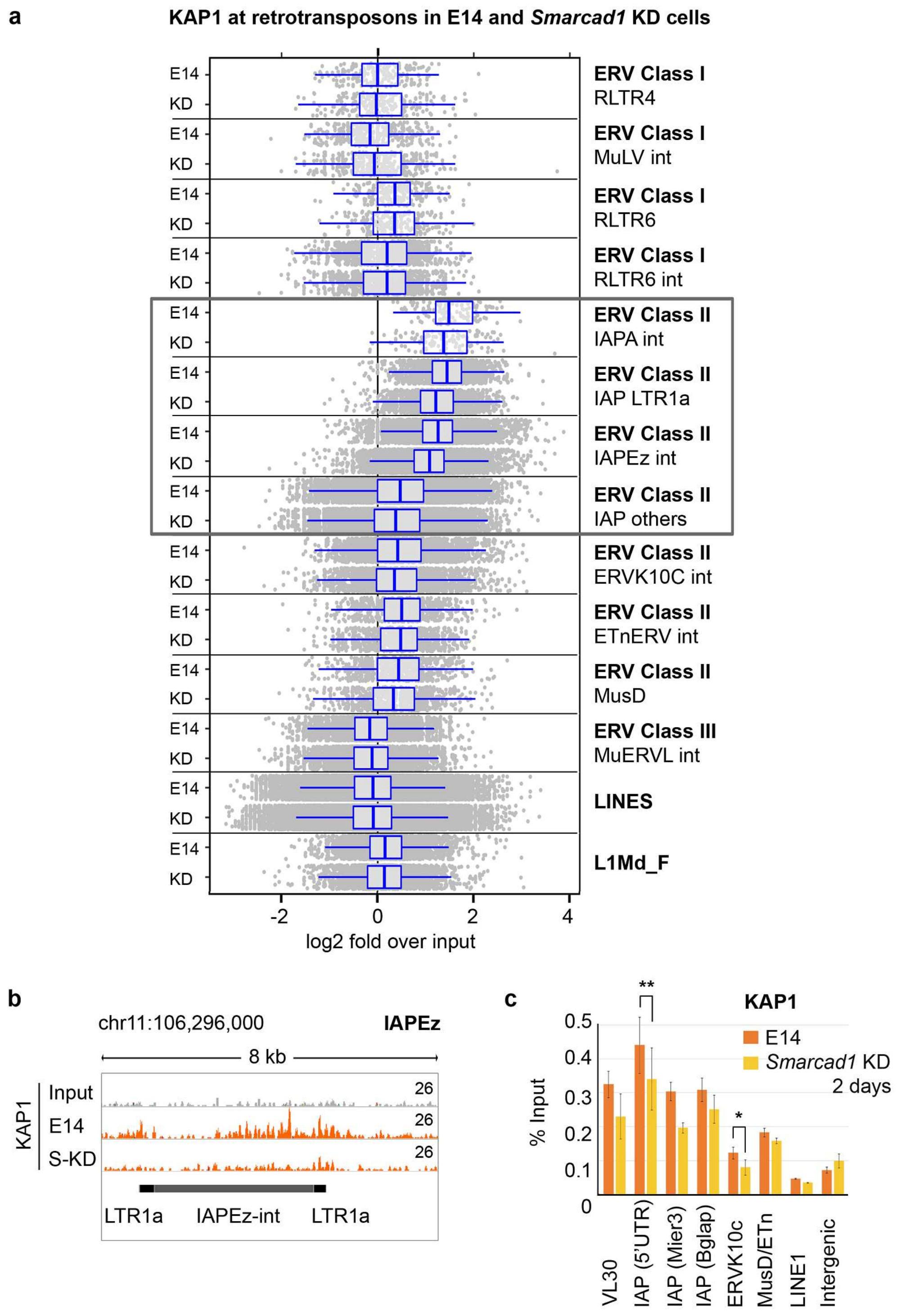

Supplementary Figure 5
Sachs et al.

Supplementary Figure 5. Related to Figure 3. KAP1 enrichment on IAP elements is reduced following knockdown of *Smarcad1.*

KAP1 ChIP-seq and ChIP-qPCR analysis in inducible *Smarcad1* knockdown E14 ESCs to measure KAP1 occupancy at retrotransposons. SMARCAD1 was depleted using doxycycline induced *Smarcad1* shRNA for 2 days as described in [2] and reduced SMARCAD1 protein levels are indicated in Figure 6c (compare lanes 1 and 2).

(a) KAP1 ChIP-seq reads were aligned to the repeat database and visualized in a combined box- and jitterplot as log2 fold ratio over input of TPM. Each dot represents a single repetitive element. In the boxplot centre lines shows the median; lower and upper box lines correspond to 25th and 75th percentiles. Repeat subfamilies were annotated according to the UCSC RepeatMasker as described in Figures 2a and Supplementary Figure 3a. A reduction in the log2 ratio was apparent at IAP elements upon *Smarcad1* KD (boxed).

(b) An IGV genome browser screenshot of an IAPEz element at chromosome 11, illustrating reduced KAP1 ChIP-seq signal upon *Smarcad1* knockdown (S-KD).

(c) A reduction of KAP1 binding upon SMARCAD1 depletion was further confirmed by ChIP-qPCR. Percent of input values are mean ± S.E. of biological triplicates (n=3). *P* values are from paired two-tailed Student's *t*-test: $*p < 0.05$, $**p < 0.01$.

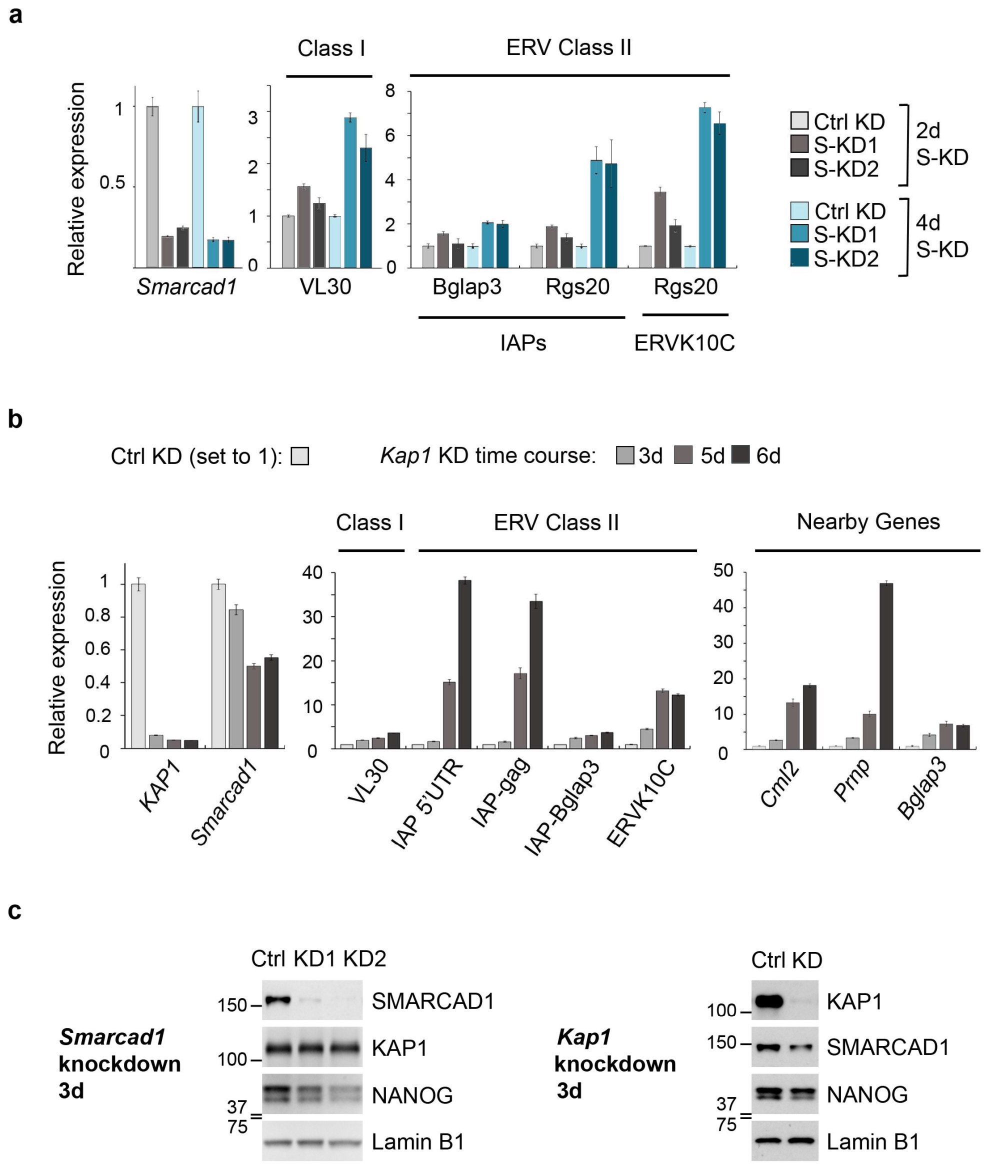

Supplementary Figure 6
Sachs et al.

Supplementary Figure 6. Related to Figure 4. The effect of *Smarcad1* or *Kap1* knockdown on the expression of ERVs.

(a) *Smarcad*1 knockdown leads to de-repression of retrotransposons. RT-qPCR analysis in E14 ESCs treated with two different *Smarcad1* shRNA constructs (S-KD1 and S-KD2) comparing expression of indicated loci following 2 or 4 days of depletion. Relative expression was normalized to the control knockdown based on one housekeeping gene. Error bars represent the mean ± S.E. of technical triplicates from one time-course experiment.

(b) The degree of transcriptional upregulation upon KAP1 depletion is time dependent. RT-qPCR analysis at indicated retrotransposons and genes in their vicinity in E14 ESCs at 3,5 or 6 days after transfection with a *Kap1* shRNA. Relative expression was normalized to the respective control knockdown based on three housekeeping genes. Error bars represent the mean ± S.E. of technical triplicates from one time-course experiment.

(c) Western blot of samples used in Figures 4a,b for RNA expression analysis, confirming that shRNA mediated depletion of SMARCAD1 and KAP1 for 3 days resulted in effective depletion of these proteins. As reported previously [2], KAP1 knockdown leads to reduced SMARCAD1 levels. The pluripotency marker NANOG is also reduced upon depletion of either protein.

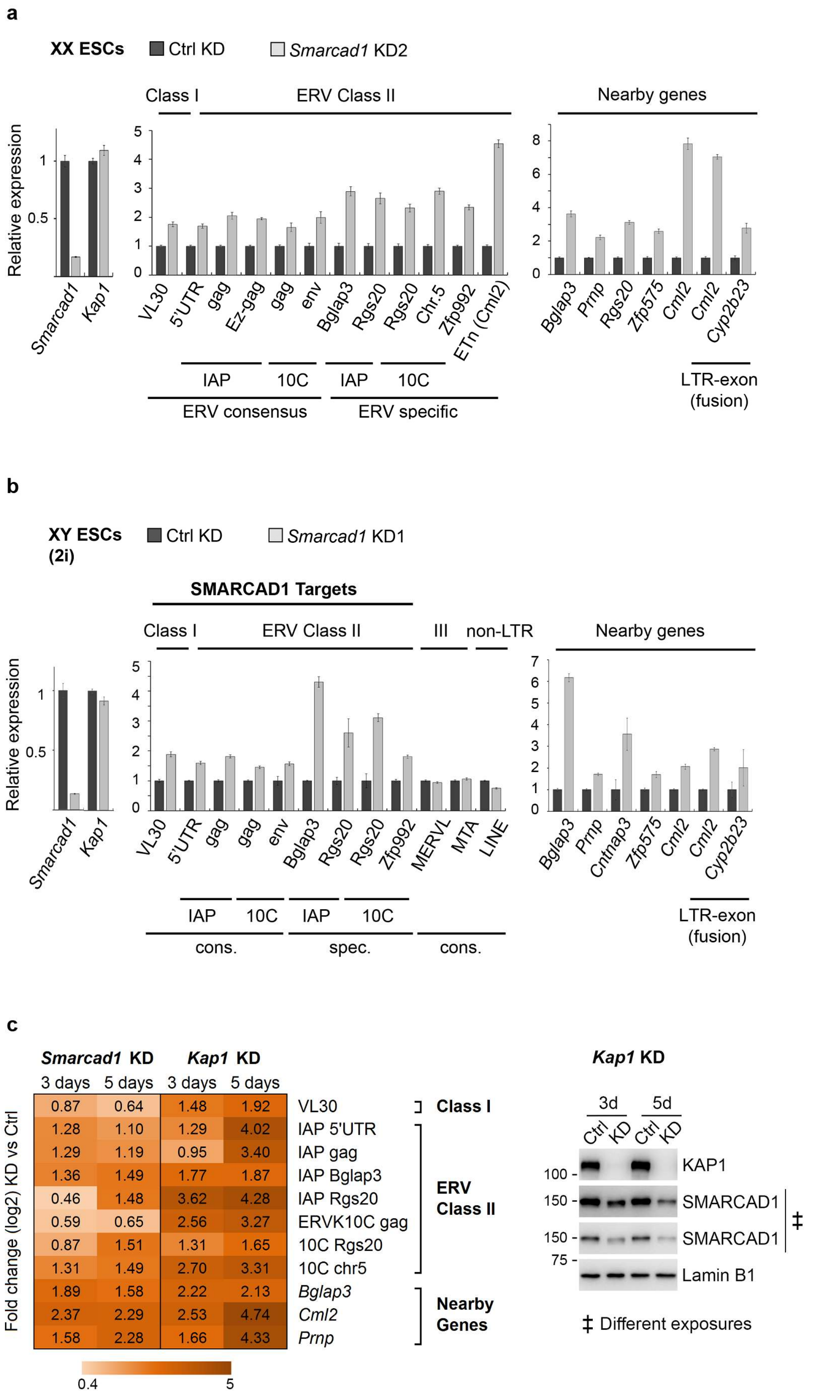

Supplementary Figure 7
Sachs et al.

Supplementary Figure 7. Related to Figure 4. The effect of *Smarcad1* knockdown on the expression of ERVs and adjacent genes.

(a and b) SMARCAD1 depletion leads to de-repression of SMARCAD1 bound retrotransposon classes and impacts neighbouring genes, including the production of chimeric transcripts containing ERV and genic sequences (LTR-exon fusion). RT-qPCR analysis at indicated loci after *Smarcad1* knockdown (KD) in (b) PGK12.1 ESCs grown in serum (4 day KD with shRNA2) and (c) E14 ESCs grown in 2i conditions (5 day KD achieved by two rounds of transfection with shRNA1). Relative expression was normalized to the respective control knockdown based on two housekeeping genes (*ATP5b* and *Gapdh*). Error bars represent the mean ± S.E. of technical triplicates. Consensus primers recognize UTR, *gag* or *envelope* consensus sequences of either IAPs or MMERVK10C elements. ERV specific primers were designed against individual copies of retrotransposons. ERV specific, gene specific primers and primers that detect LTR-fusion products are depicted in genome browser screenshots in Supplementary Figure 8.

(c) Transcription changes at ERVs and nearby genes induced by depletion of either SMARCAD1 (*Smarcad1* KD) or both KAP1 and SMARCAD1 (*Kap1* KD). E14 ESCs were transfected with shRNAs targeting either *Smarcad1*, or *Kap1* or control shRNAs (Ctrl). Left panel; Heat map depicts the RNA levels of ERVs and nearby genes in log2 fold change relative to the control KD measured by RT-qPCR 3 days and 5 days post transfection (n=1). Right panel; Western blot analysis of 3 day and 5 day *Kap1* KD cells with indicated antibodies. Different exposures of SMARCAD1 highlight the progressive decline in SMARCAD1 levels.

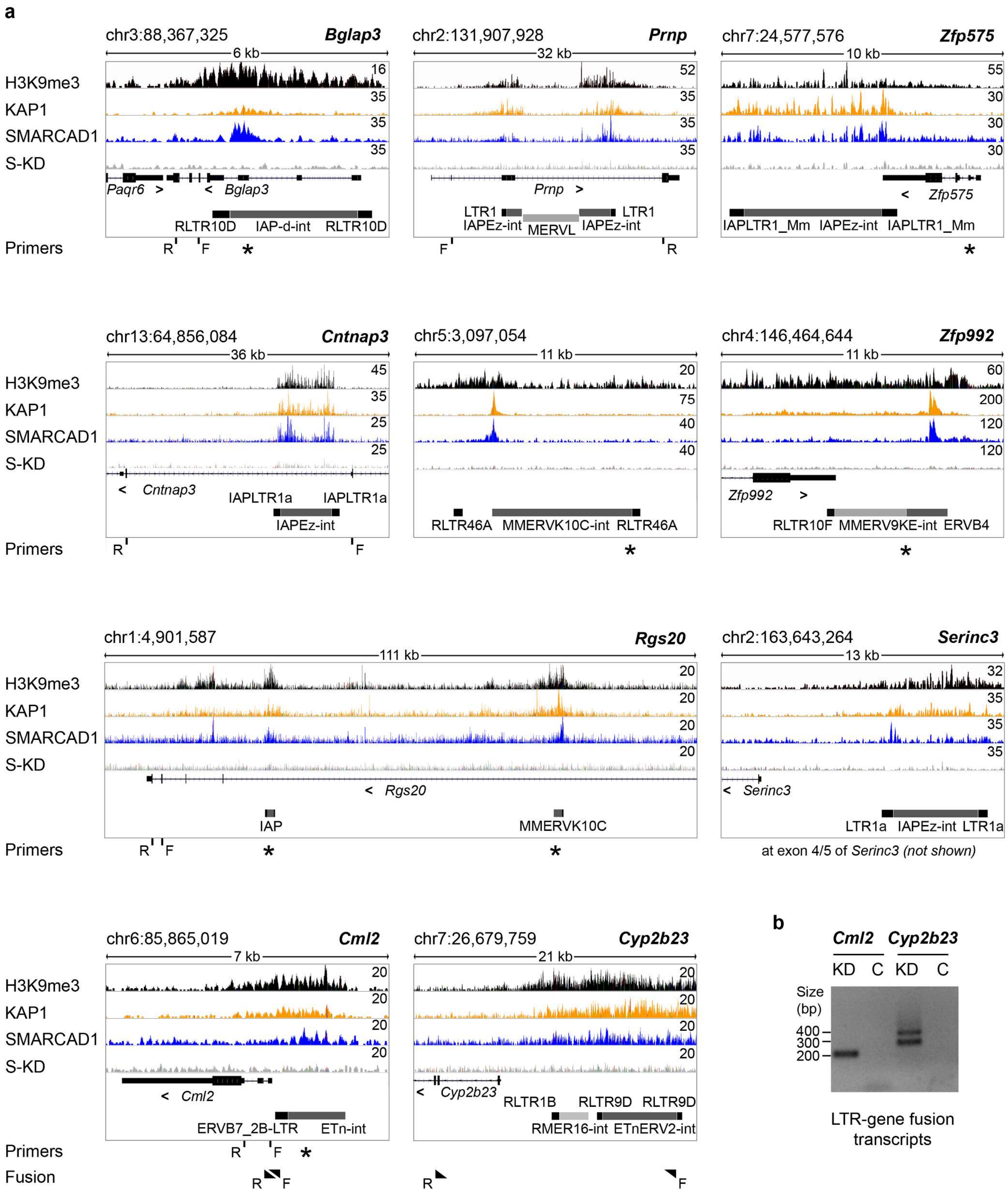

Supplementary Figure 8
Sachs et al.

Supplementary Figure 8. Related to Figure 4 and Supplementary Figures 6,7. H3K9me3, SMARCAD1 and KAP1 binding at ERVs and detection of ERV-gene fusion transcripts.

(a) Integrative Genome Browser (IGV) view of genomic regions containing ERVs regulated by SMARCAD1. The scale varies between 6 and 111 kb. The orientation of the genes is indicated with an arrowhead. ChIP-seq signal of SMARCAD1 in Ctrl and *Smarcad1* knockdown (S-KD) mESCs is depicted, along with the primers used to detect expression upon knockdown of *Smarcad1*/*Kap1* (Figure 4). Forward and reverse primers are indicated with F and R; amplicons too small to depict on the scale are indicated by an asterix. The ChIP-seq signals of H3K9me3 (this study) and KAP1 [1] are also shown.

(b) *Smarcad1* knockdown (KD) leads to production of chimeric transcripts of LTR elements of promoter proximal ERVs and genes nearby. An agarose gel showing the RT-qPCR products generated in Supplementary Figure 6b; C refers to 'no RT' negative control sample. *Cml2* primers designed to recognize an LTRERVB27_2B element 5' to the annotated TSS and downstream sequences within exon 1 of the *Cml2* gene (see panel a) amplify a 217 bp product expected in the presence of transcriptional fusion. *Cyp2b23* primers map 5' to the annotated TSS in an ETnERV2/MusD element and in exon 2 (see panel a) [3]. Fusion products of 290 and 390 bp are detected, chimeric products of this size have previously been characterized upon *Setdb1* KO at this locus [3].

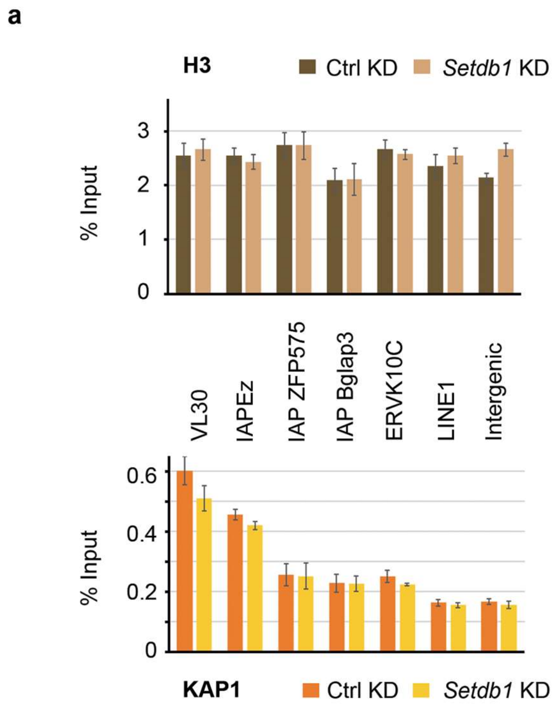

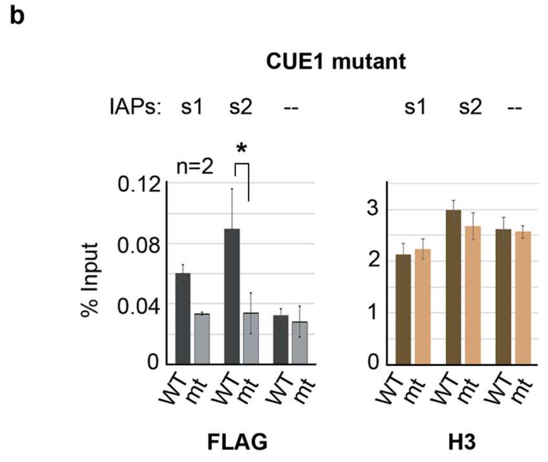

Supplementary Figure 9
Sachs et al.

Supplementary Figure 9. Related to Figure 5. SMARCAD1 binding to ERVs is not dependent on SETDB1 but requires interaction with KAP1.

(a) Characterization of H3 and KAP1 occupancy over retrotransposons after transient depletion of SETDB1 by ChIP-qPCR. Chromatin analysed corresponds to samples from Figure 5a. Data are representative of two immunoprecipitations and error bars denote the mean ± S.E. of technical triplicates.

(b) Stable association of SMARCAD1 with IAP elements depends on an intact CUE1 domain in SMARCAD1. FLAG ChIP-qPCR reveals that binding of tagged wild-type SMARCAD1 protein (WT) is disrupted when SMARCAD1 is mutated in the KAP1 interaction domain (CUE1 mt; F168K, L195K). IAP element s1 corresponds to Bglap3, s2 corresponds to Mier3. Analysis of an intergenic region without a known IAP element is shown as a control. A representative example of corresponding H3 ChIP (n=2) over these sites is also shown with error bars depicting the mean ± S.E. of technical triplicates. FLAG ChIP data are the mean ± S.E. of biological triplicates (n=3) with the exception of s1 (*Bglap3*), which represents the mean ± S.E. of biological duplicates and thus has been excluded from statistical analysis. *P* values are from paired two-tailed Student's *t*-test: $*p < 0.05$, $**p < 0.01$, $***p < 0.001$.

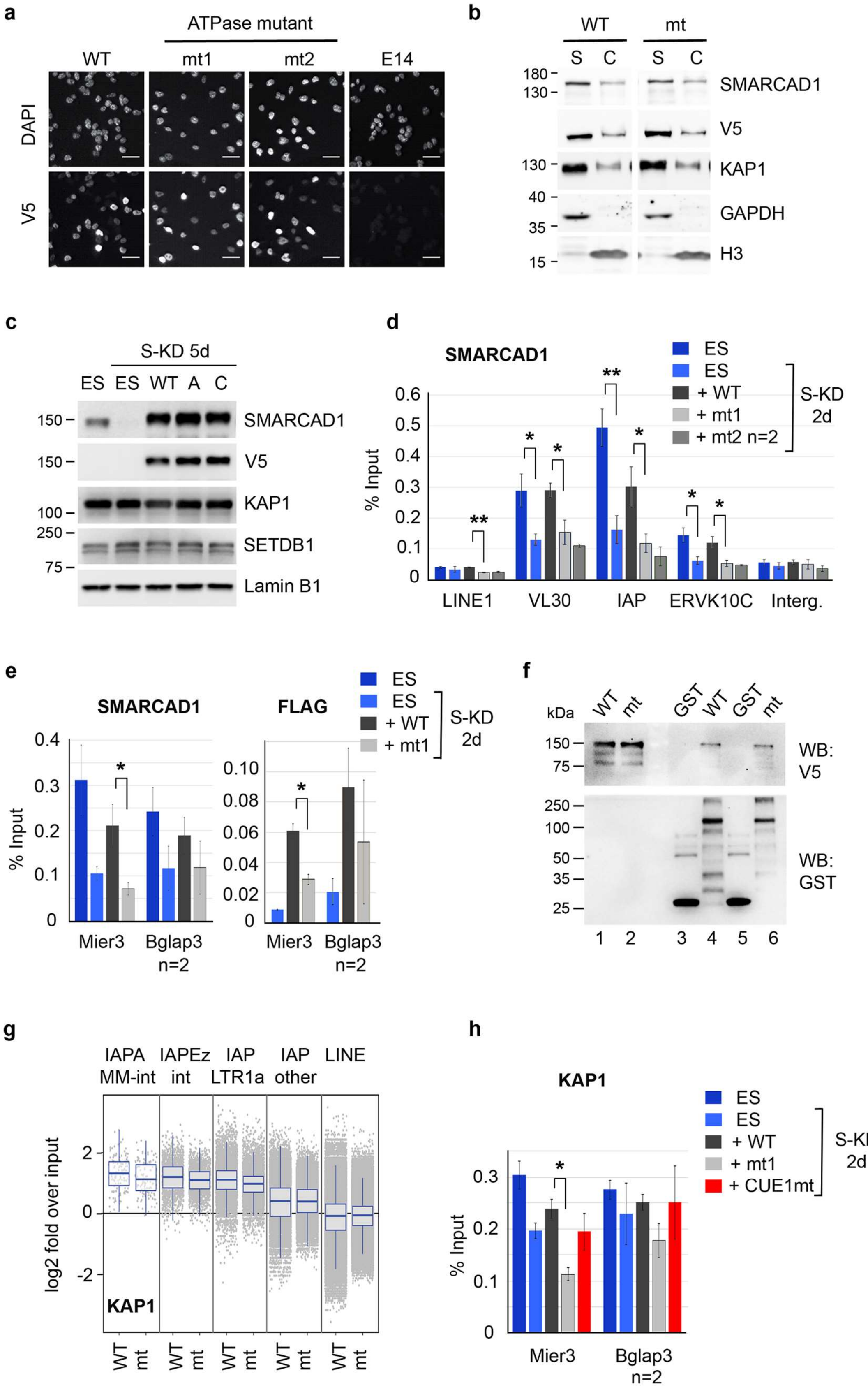

Supplementary Figure 10
Sachs et al.

Supplementary Figure 10. Related to Figure 6. An active ATPase domain is required for SMARCAD1 function at ERVs.

(a) Nuclear localization of tagged SMARCAD1 proteins in ESCs described in Figures 6b and 6c. Immunofluorescence analysis of cells expressing WT protein (WT) and ATPase mutant (mt1, mt2) using an antibody against the C-terminal V5 tag. ATPase mutants 1 and 2 refer to two independent clones. The right panel depicts untransfected cells (E14) and serves as a negative control. Analysis was performed after depletion of endogenous SMARCAD1 with a 48 hour doxycycline treatment. Nuclei were counterstained with DAPI. Scale bar represents 20 μm.

(b) SMARCAD1 ATPase mutant behaves as the wildtype protein in cellular fractionation experiments. Western blot analysis of ESCs expressing tagged SMARCAD1, either WT or ATPase mutant (mt), fractionated into chromatin enriched (C) and soluble fractions (S). Like KAP1, both WT and mutated SMARCAD1 are predominantly in the soluble fraction. Extract amounts corresponding to the same number of cells were loaded on the same gel and probed with indicated antibodies.

(c) Western blot of samples used in Figure 6d for expression analysis. Level of SMARCAD1 knockdown is shown with a SMARCAD1 antibody by comparing lane 1 with lane 2. A V5 antibody was used to confirm that all tagged SMARCAD1 protein variants (WT: wild-type; A: ATPase mt; C: CUE1 mt) were expressed to similar levels. SETDB1 and KAP1 steady state levels were not affected in either condition. Lamin B1 serves as a reference across cell lines.

(d, e and h) ChIP was performed in the same cells as in Figure 6 after 2 day dox treatment. The level of enrichment of SMARCAD1, KAP1 and FLAG-tagged proteins was analysed by qPCR over representative transposable elements of class I (VL30) and class II (IAP 5'UTR and MMERVK10C in (d); IAPs *Mier3* and *Bglap3* in (e) and (h), respectively). LINE1 retrotransposon and intergenic sequences serve as negative controls. The SMARCAD1 antibody precipitates both endogenous and exogenous SMARCAD1. Percent of input values are mean ± S.E. of biological triplicates (n=3) with the following exceptions: ATPase mutant 2 chromatin and the Bglap3 locus. These were not examined in a third experiment, excluding them from statistical analysis. Paired two-tailed Student's *t*-test was performed otherwise (*$p < 0.05$, **$p < 0.01$, ***$p < 0.001$).

(f) The ATPase mutation in SMARCAD1 does not alter binding to KAP1. *In vitro* transcribed/translated V5-SMARCAD1 (lane 1: WT protein; lane 2: ATPase mutant protein) was employed in GST pulldown assays with either GST (26 kDa; lanes 3,5) or GST-KAP1ΔPB (100 kDa; lanes 4,6). Top, detection of bound proteins by Western blot (WB) with anti-V5 antibody. Bottom, GST fusion proteins detected with an anti-GST antibody indicating that comparable amounts of bait proteins were used in each binding reaction.

(g) Levels of KAP1 binding at IAPs in ESCs depleted for endogenous SMARCAD1 expressing FLAG-SMARCAD1 proteins, either the wild-type protein (WT) or the ATPase mutant SMARCAD1 (mt). ChIP-seq data are presented as in Figure 6e, a combined box- and jitterplot depicting the log2 fold ratio over input of TPM.

Supplementary Figure 11: Source Data.

Fig.1a

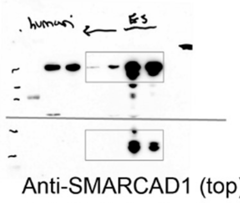

Anti-SMARCAD1 (top)
Anti-Oct4 (bottom)

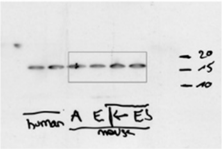

Anti-H3

Fig.1b

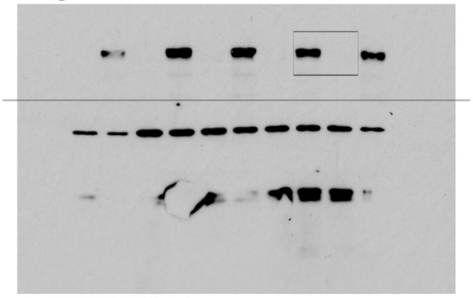

Anti-SMARCAD1
Anti-Lamin B1 (exposure not used)
Anti-NANOG (exposure not used)

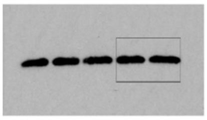

Anti-H3

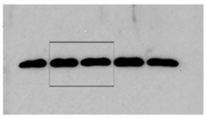

Anti-H3K9me3

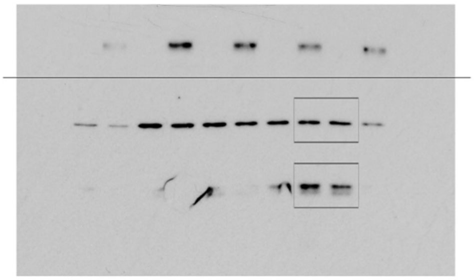

Anti-SMARCAD1 (exposure not used)
Anti-Lamin B1
Anti-NANOG

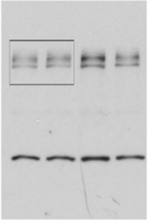

Anti-G9a
Anti-Lamin B1
(not used)

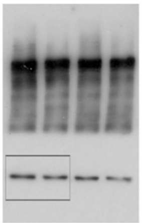

Anti-SETDB1
(exposure not used)
Anti-HDAC1

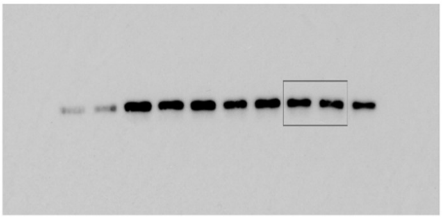

re-probed with anti-KAP1

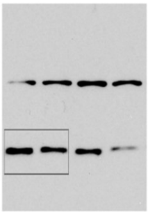

Anti-Lamin B1
(not used)
Anti-Suv39h1

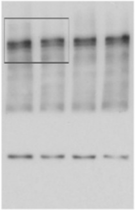

Anti-SETDB1
Anti-HDAC1
(exposure not used)

molecular weight markers indicated

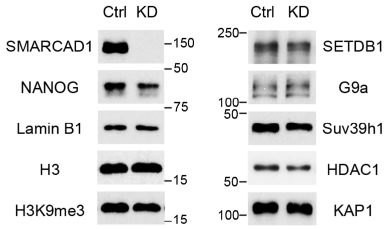

Supplementary Figure 11
Sachs et al.

Fig.1g

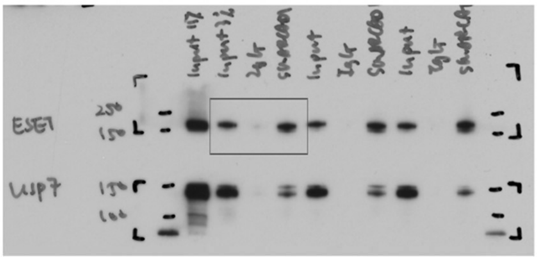

Anti-SETDB1

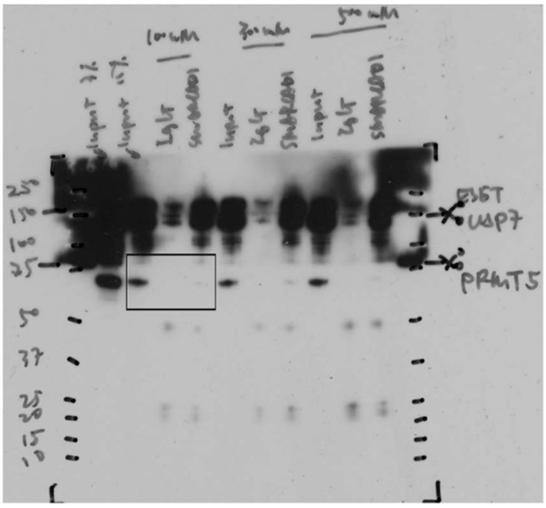

Anti-PRMT5

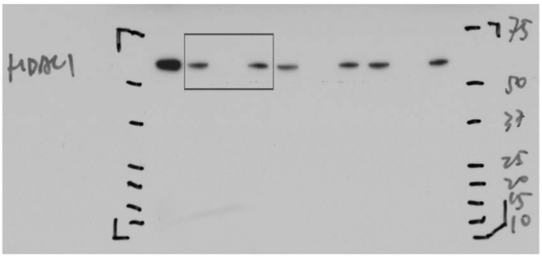

Anti-HDAC1

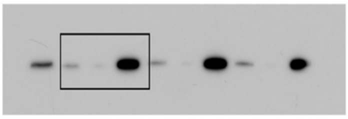

Anti-SMARCAD1

Fig.5a

Gel1:

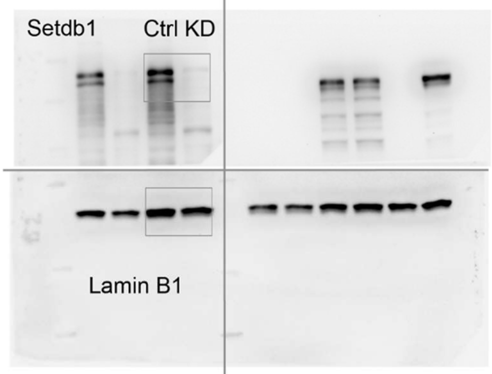

Gel2:

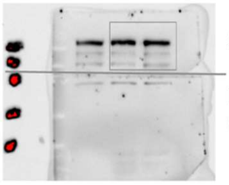

SMARCAD1

Lamin B1
(not shown)

Supplementary Figure 11
Sachs et al.

Fig.5b

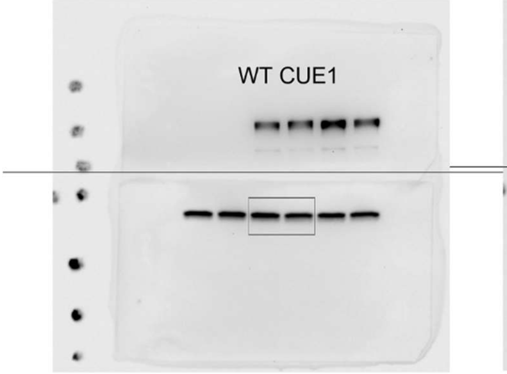

Anti-V5 (exposure not used)
Anti-Lamin B1

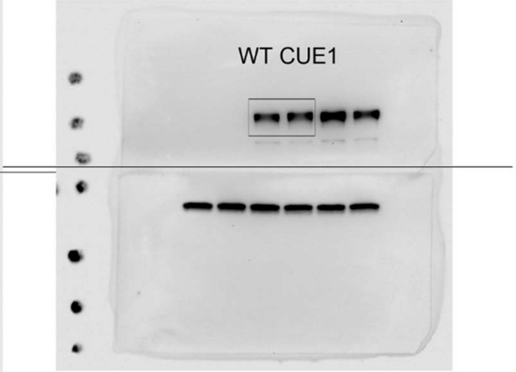

Anti-V5
Anti-Lamin B1 (exposure not used)

Fig.6c

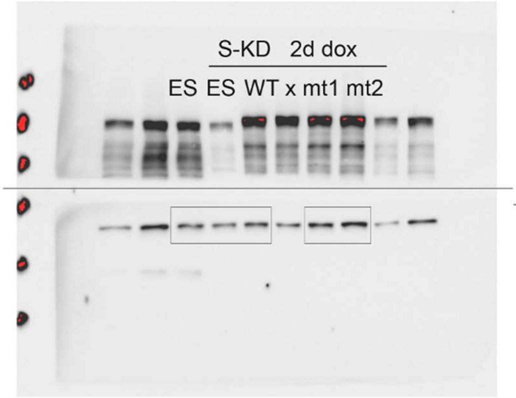

Anti-SMARCAD1 (exposure not used)
Anti-Lamin B1

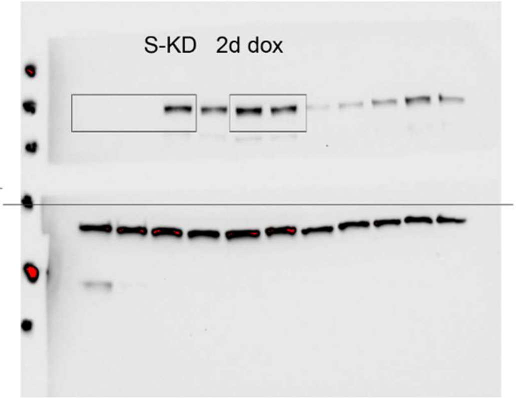

Anti-V5
Lamin B1 (not used)

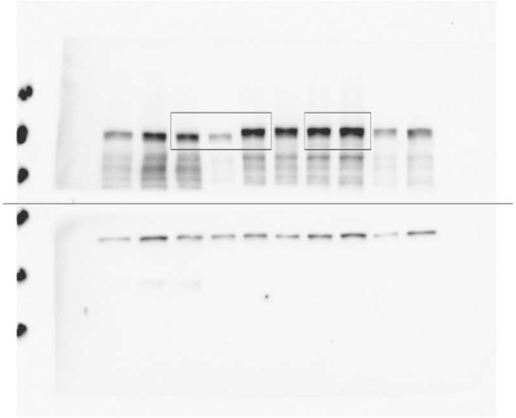

Anti-SMARCAD1
Anti-Lamin B1 (exposure not used)

Supplementary Figure 11
Sachs et al.

Fig.6c continued

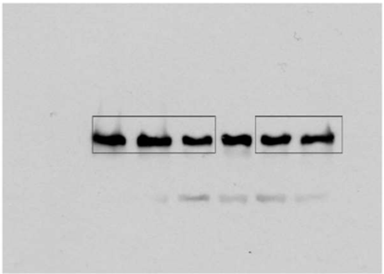

Anti-KAP1

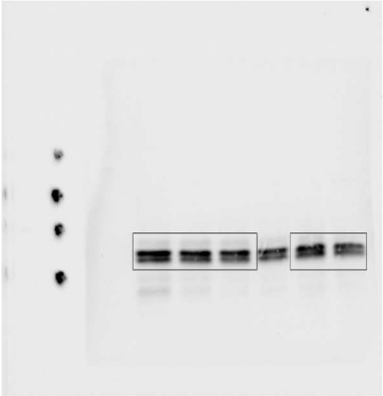

Anti-NANOG

Fig.6g

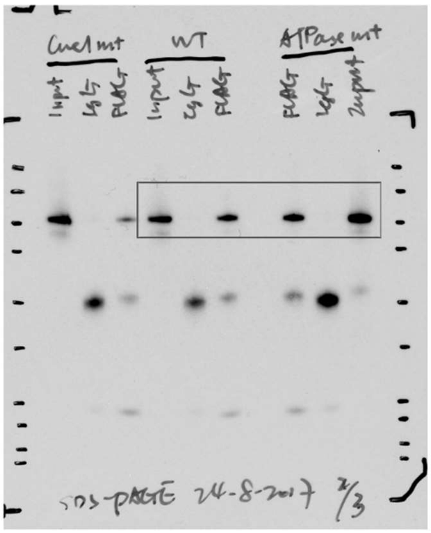

Anti-KAP1

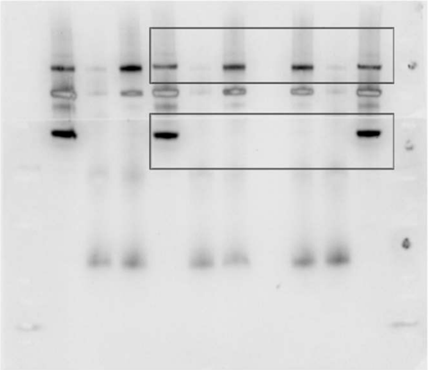

Re-probed:
Anti-FLAG
Anti-PRMT5

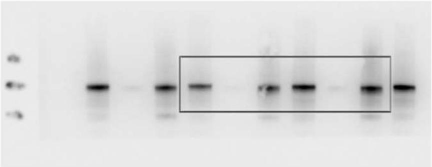

Anti-SMARCAD1

Supplementary Figure 11
Sachs et al.

Supplementary Fig.1f

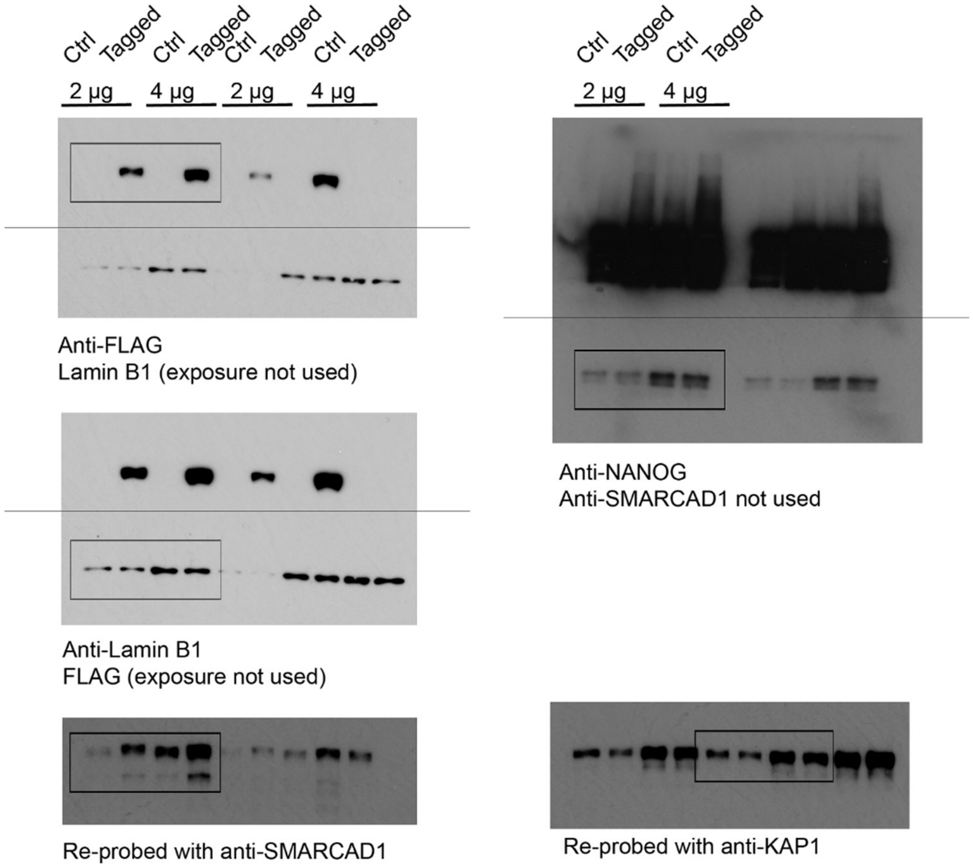

Supplementary Fig.2h

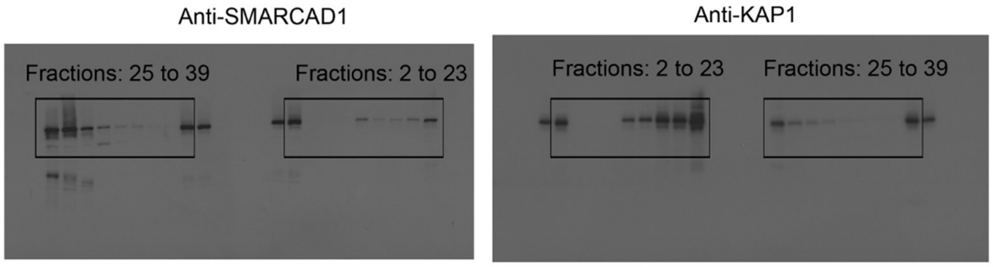

Same membranes, probed anti-SMARCAD1 first, then stripped and probed anti-KAP1.
Developed on film.

Supplementary Figure 11
Sachs et al.

Supplementary Fig.2h continued

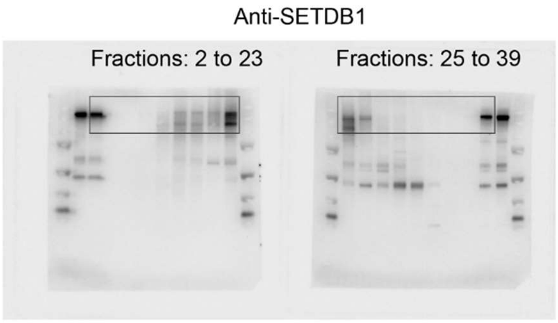

Supplementary Fig. 4f

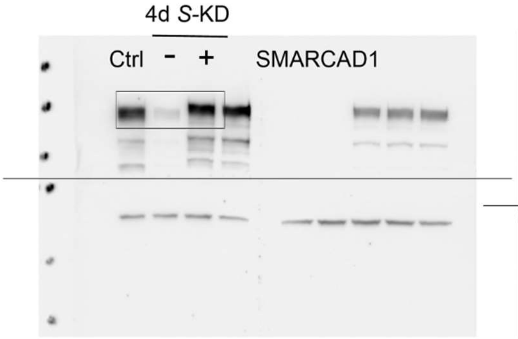

Anti-SMARCAD1 / Anti-V5 (not used)
Anti-Lamin B1 (not used)

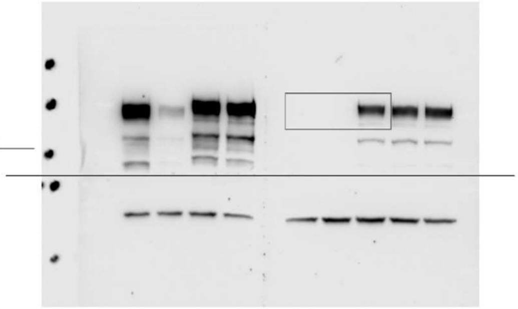

Anti-SMARCAD1 (not used) / Anti-V5
Anti-Lamin B1 (not used)

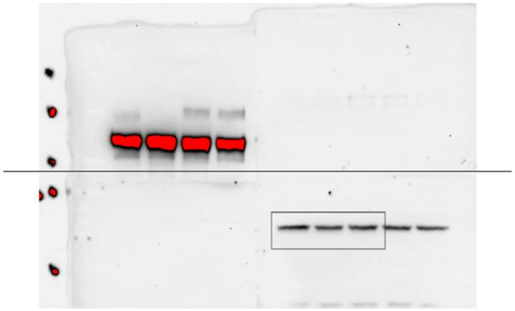

Anti-KAP1 (exposure not used)
Anti-Lamin B1

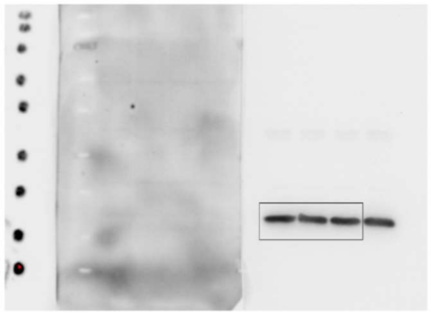

Anti-H3

Supplementary Figure 11
Sachs et al.

Supplementary Fig. 4f continued

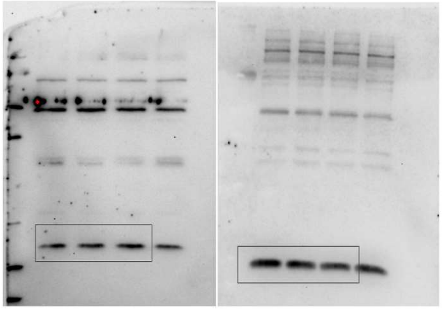

Anti-H3K9me3 Anti-H4K20me3

Supplementary Fig.6c

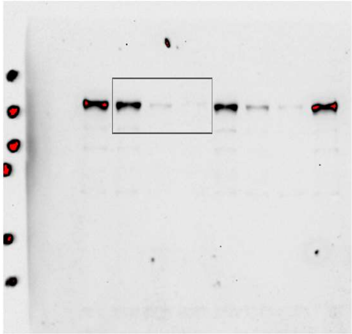

Anti-SMARCAD1

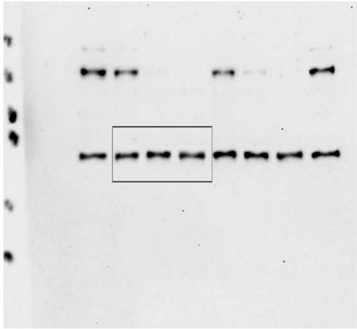

Loading ctrl: anti-Lamin B1
(not used)

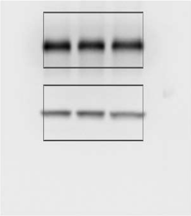

Anti-KAP1
Anti-Lamin B1

Re-probed:

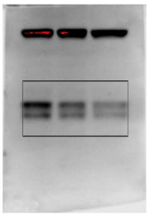

Anti-Lamin B1
(not used)

Anti-NANOG

Supplementary Figure 11
Sachs et al.

Supplementary Fig. 6c continued

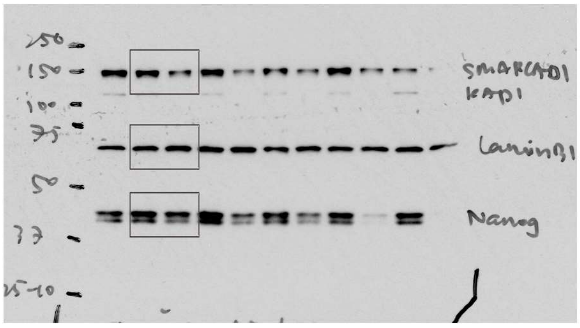

Anti-SMARCAD1
Anti-Lamin B1
Anti-NANOG

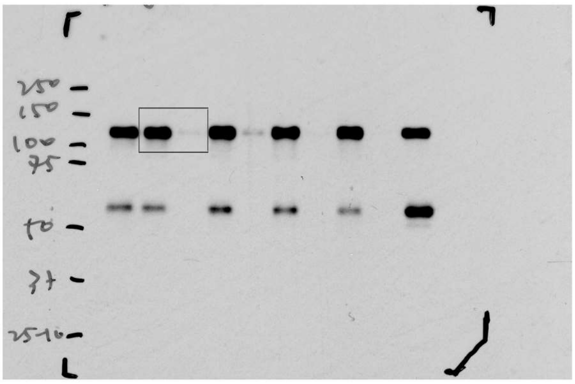

Re-probed:
Anti-KAP1

Supplementary Fig. 7c

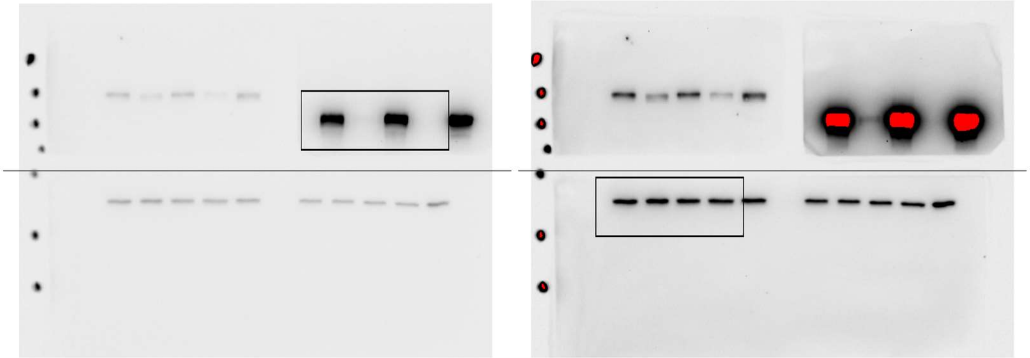

SMARCAD1 (not used)
Lamin B1 (not used)

KAP1
Lamin B1(not used)

SMARCAD1 (not used)
Lamin B1

KAP1 (not used)
Lamin B1(not used)

Supplementary Figure 11
Sachs et al.

Supplementary Fig. 7c continued

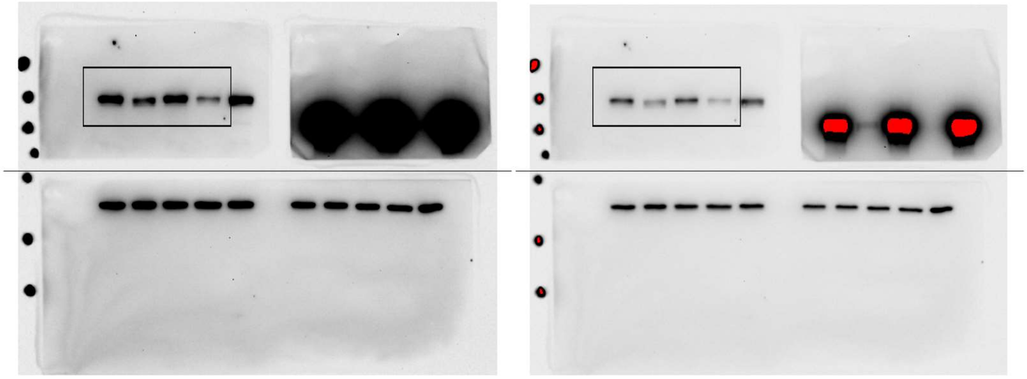

SMARCAD1
Lamin B1 (not used)

KAP1 (not used)
Lamin B1(not used)

SMARCAD1
Lamin B1 (not used)

KAP1 (not used)
Lamin B1(not used)

Supplementary Fig. 8b

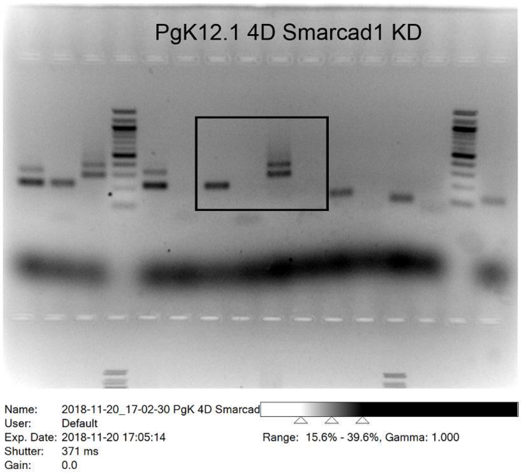

Supplementary Figure 11
Sachs et al.

Supplementary Fig. 10b

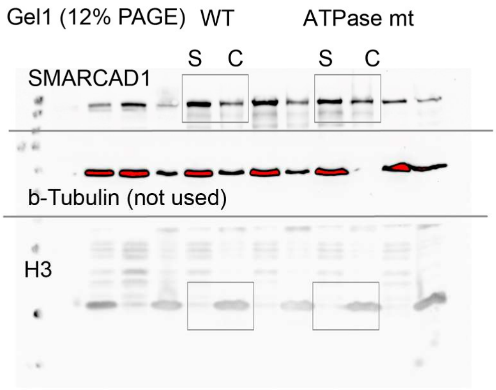

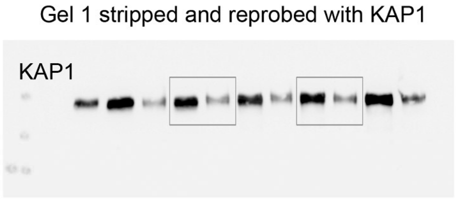

Supplementary Fig. 10b continued

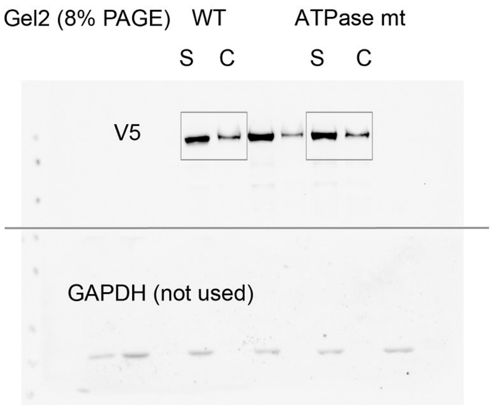

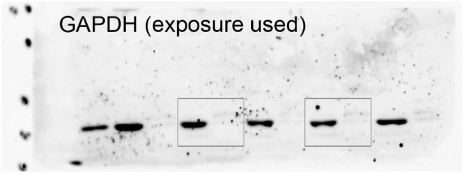

Supplementary Figure 11
Sachs et al.

Supplementary Fig. 10c

S-KD 5d

ES ES WT A C

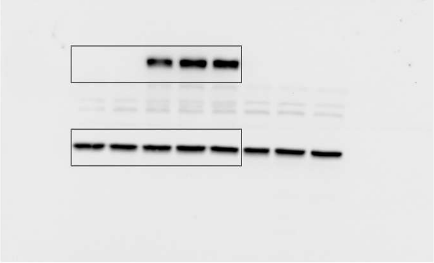

Anti-V5
Anti-Lamin B1

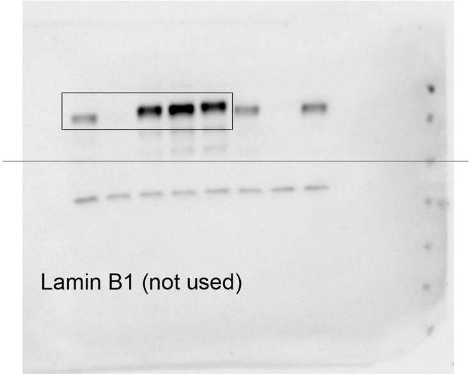

Anti-SMARCAD1

S-KD 5d

ES ES WT A C

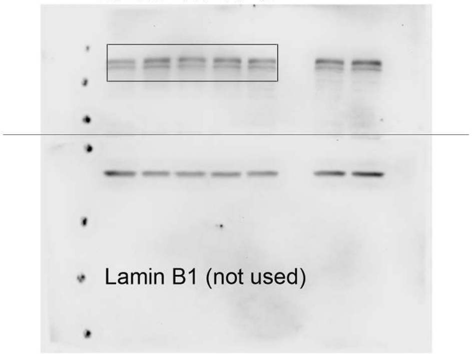

Anti-SETDB1

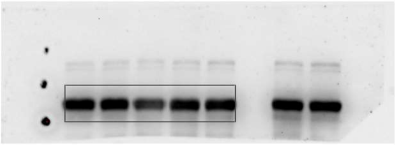

Reprobed with Anti-KAP1

Supplementary Fig. 10f

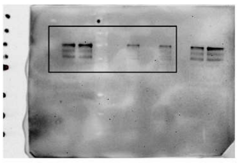

anti-V5

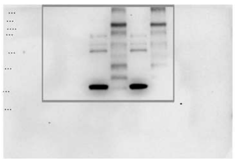

anti-GST

Uncropped Western blot.

Supplementary Figure 11. Source Data. Uncropped Western blot and gel images. Cropped regions used in the main and supplementary figures are outlined.

Supplementary Table 1 **Summary of SMARCAD1 & FLAG-SMARCAD1 & H3K9me3 and KAP1 ChIP-seq data**

ChIP Seq	endogenous SMARCAD1	SMARCAD1 knockdown	FLAG SMARCAD1	H3K9me3	FLAG SMARCAD1			KAP1			
ESCs	PGK12.1	PGK12.1	PGK12.1	PGK12.1	E14 + dox	E14 + dox	E14 + dox	E14	E14 + dox	E14 + dox	E14 + dox
						FLAG-WT SMARCAD1	FLAG-ATPase mt			FLAG-WT SMARCAD1	FLAG-ATPase mt
Cross-linking	double	double	double	double	double	double	double	double	double	double	double
ChIP antibody	SMARCAD1	SMARCAD1	FLAG	H3K9me3	FLAG	FLAG	FLAG	KAP1	KAP1	KAP1	KAP1
Effective reads	7.93E+07	6.52E+07	5.05E+07	5.47E+07	3.49E+07	8.24E+07	7.24E+07	4.34E+07	3.17E+07	2.56E+07	3.52E+07
Used background	IgG WT	IgG KD	FLAG Ab	IgG WT							
	Input WT	Input KD	Input of Ctrl	Input WT	Input	Input WT SMARCAD1	Input ATPase mt	Input	Input	Input WT SMARCAD1	Input ATPase mt
Effective background reads	6.71E+07	6.44E+07	4.66E+07	6.71E+07							
	7.96E+07	6.37E+07	6.22E+07	7.96E+07	4.81E+07	5.24E+07	6.85E+07	4.97E+07	4.81E+07	5.24E+07	6.85E+07
Peak caller	MACS	MACS	MACS	SICER	MACS	MACS	MACS	MACS	MACS	MACS	MACS
unfiltered peaks	15418	287	8786	55183	3311	8728	5119	17592	15225	19090	9467
filtered peaks *	5727	63	3317	10849	94 **	2816	1081	5360	1404	767	1153

An optimised double crosslinking ChIP-seq protocol (DSG+FA) allows for robust enrichment of SMARCAD1 in mESCs. Comparable results were obtained with an exogenous expression approach using FLAG-tagged SMARCAD1 and with ChIP of endogenous SMARCAD1. "Effective reads" are reads aligned to mm10 using Bowtie2 followed by removal of duplicate reads. Only peaks that showed a strong enrichment compared to their background (IgG, FLAG antibody or Input) were kept as "filtered peaks". "Effective background reads" refers to the number of aligned, de-duplicated reads for each background category. * If more than one background was available for a sample only those peaks were kept that showed a strong enrichment compared to both backgrounds. ** Unspecific FLAG antibody background; peaks from FLAG ChIP in in E14 ESCs overlapping with a least one of these peaks were removed during filtering. Antibodies used: SMARCAD1 PAB15737, Abnova; H3K9me3 ab8898 (abcam); FLAG F1804 (Sigma) and KAP1 22553 (abcam)

Supplementary Table 2 List of antibodies used in this study

Antibody	Antigen	Source of Antibody	Method		
			IF	ChIP	Western
SMARCAD1 HPA016737	Rabbit	Sigma	1/80	3ug	1/1,000
SMARCAD1 PAB15737	Rabbit	Abnova		3ug	1/2,500
SMARCAD1 A301-593A	Rabbit	Bethyl		3ug	1/1,000
SMARCAD1 A301-592A	Rabbit	Bethyl		3ug	
SMARCAD1 ab67548	Rabbit	Abcam		3ug	
SMARCAD1 anti-CUE	Rabbit	Rowbotham et al. 2011	1/400	3ul	1/5,000
Lamin B1 ab16048	Rabbit	Abcam			1/10,000
KAP1 ab22553	Mouse	Abcam		3ug	1/10,000
IgG kCH-504 C15410206	Rabbit	Diagenode		3ug	
IgG I5381	Mouse	Sigma		3ug	
FLAG F3165	Mouse	Sigma			1/5,000
FLAG F1804	Mouse	Sigma	1/400	3ug	1/4,000
V5 R960-25	Mouse	Thermo Fisher Scientific			1/5,000
H3 ab1791	Rabbit	Abcam		3ug	1/10,000
H39me3 ab8898	Rabbit	Abcam		3ug	
H3K9me3 07-523	Rabbit	Milipore			1/5,000
H3K9me3 39161	Rabbit	Active-Motif			1/1,000
H4K20me3 9053	Rabbit	Abcam		3ug	1/1,000
SETDB1 11231-1-AP	Rabbit	Proteintech		3ug	
PRMT5 07-405	Rabbit	Millipore			1/5,000
HDAC1 05-100	Mouse	Millipore			1/2,500
OCT4 sc-5279	Mouse	Santa Cruz			1/1,000
NANOG A300-397A	Rabbit	Bethyl	1/200		1/10,000
G9a PP-A8620A-00	Mouse	R&D			1/2,000
SUV39H1 8729	Rabbit	Cell signaling			1/1,000
SETDB1 sc-66884	Rabbit	Santa Cruz			1/1,000
GST sc-138	Mouse	Santa Cruz			1/10,000
GAPDH sc-25778	Rabbit	Santa Cruz			1/1,000

Supplementary Table 3 List of PCR primers used in this study

Target	Sequence (5'-3')	Application	Genomic copy number in silico PCR	Amplicon size, bp	Reference
**MLV_F**	AAACCCCGGAAGAAAGAGAG	ChIP	61	80	M. Branco
**MLV_R**	CTGCTCATCCTCTGCCCTAC	ChIP			
**VL30 pro (PBS-UTR)_F**	TGGGGGCTCGTCCGGGAT	ChIP/RT	15	~ 78	Wolf et al., 2015
**VL30 pro (PBS-UTR)_R**	ATTACCAAGCGACAGAACTTACC	ChIP/RT			
**IAPc* (5'UTR)_F**	CGGGTCGCGGTAATAAAGGT	ChIP	1038	91	Rowe et al., 2010
**IAPc* (5'UTR)_R**	ACTCTCGTTCCCCAGCTGAA	ChIP			
**IAPs* (MIER3)_F**	GAACCTCTTGCCTCTTCCCC	ChIP	1	196	this study
**IAPs* (MIER3)_R**	GCTTGGGAGATGGATCCCAG	ChIP			
**IAPs* (Bglap3)_F**	AGGTGTTGCAGAGGTTTTGG	ChIP/RT	1	89	Ecco et al., 2016
**IAPs* (Bglap3)_R**	AATATCGGACACAGGGCAAG	ChIP/RT			
**IAPs* (ZFP575)_F**	TGAGCCTCTGTGTGGGTCCTA	ChIP	1	67	Rowe et al., 2013
**IAPs* (ZFP575)_R**	TGATTAAGAGCACTTGTTGCTTAGC	ChIP			
**IAPez_F**	GCTCCTGAAGATGTAAGCAATAAAG	ChIP	688	~143	Liu et al., 2014
**IAPez_R**	CTTCCTTGCGCCAGTCCCGAG	ChIP			
**IAPez gag_F**	CACGCTCCGGTAGAATACTTACAAAT	ChIP/RT	844	96	Sharif et al., 2016
**IAPez gag_R**	CCTGTCTAACTGCACCAAGGTAAAAT	ChIP/RT			
**IAPU3_F**	CGAGGGTGGTTCTCTACTCCAT	ChIP	190	88	Rowe et al., 2010
**IAPU3_R**	GACGTGTCACTCCCTGATTGG	ChIP			
**IAPpol_F**	CTTGCCCTTAAAGGTCTAAAAGCA	ChIP	490	77	Rowe et al., 2010
**IAPpol_R**	GCGGTATAAGGTACAATTAAAAGATATGG	ChIP			
**IAP1pol_F**	TGGCCATACCCCAAAGATAA	ChIP	36	119	Fasching et al., 2015
**IAP1pol_R**	CCAGTTTACTGGGGCTGGTA	ChIP			

**IAPgag_F**	AATCTCAGAACCGCTCCATGA	ChIP/RT	447	77	Rowe et al., 2010
**IAPgag_R**	TTTCTTAAAATGCCCAGGCTTT	ChIP/RT			
**Etn/Musd_F**	GATTGGTGGAAGTTTAGCTAGCAT	ChIP	129	149	Fasching et al., 2015
**Etn/Mud_R**	TAGCATTCTCATAAGCCAATTGCAT	ChIP			
**MMERKV10C_F**	TTCGCCTCTGCAATCAAGCTCTC	ChIP	79	~137	Liu et al., 2014
**MMERKV10C_R**	TCGCTCRTGCCTGAAGATGTTTC	ChIP			
**MERVL_F**	CTTCCATTCACAGCTGCGACTG	ChIP	512	155	Liu et al., 2014
**MERVL_R**	CTAGAACCACTCCTGGTACCAAC	ChIP			
**L1_F**	TTTGGGACACAATGAAAGCA	ChIP	1426	155	Fadloun et al., 2013
**L1_R**	CTGCCGTCTACTCCTCTTGG	ChIP			
**L1MdF_F**	GCATCTCTGGGGTGAGCTAG	ChIP	1	148	Castro-Diaz et al., 2014
**L1MdF_R**	AAAAGGGTGCTGCCTCAGAA	ChIP			
**Intergenic_F**	CAGCATTCCAGGAGGTTAGC	ChIP	1	147	Karnowski et al, 2008
**Intergenic_R**	GTGCCTCATGTGCAGTCAGT	ChIP			
**Ezr1_F**	GGCCCCGTAACTGCTCTTTA	ChIP	1	199	Dong et al., 2018
**Ezr1_R**	AGTATAAGACGCTGCGGCAA	ChIP			
**Ezr2_F**	TCCCTCCTGCACGTGGTAAT	ChIP	1	157	this study
**Ezr2_R**	AAGGGTCCTCACTTGACCAG	ChIP			
**Ezr3_F**	ATAGGGGCGAGTAGCCATTG	ChIP	1	71	this study
**Ezr3_R**	GTGAAGGCGTTTGTTGGACTC	ChIP			
**Atp5b_F**	GGCCAAGATGTCCTGCTGTT	RT	1	106	Wossidlo et al., 2011
**Atp5b_R**	GCTGGTAGCCTACAGCAGAAGG	RT			
**Hspcb_F**	GCTGGCTGAGGACAAGGAGA	RT	1	93	Wossidlo et al., 2011
**Hspcb_R**	CGTCGGTTAGTGGAATCTTCA	RT			
**Gapdh_F**	TCCATGACAACTTTGGCATTG	RT	1	72	Rowe et al., 2010
**Gapdh_R**	CAGTCTTCTGGGTGGCAGTGA	RT			

**Rex1_F**	CGATGCTGGAGTGTCCTCAAG	RT	1	113	Ficz et al., 2011
**Rex1_R**	GCCACACTCTGCACACACGT	RT			
**Smarcad1_F**	TTCCTGGCATACCTCTTTC	RT	1	155	Dong et al., 2018
**Smarcad1_R**	ATTTGCTTACGCTCTTCTTG	RT			
**Smarcad1_3UTR_F**	GGGGGTCAGTTCCTTCTTTC	RT	1	126	this study
**Smarcad1_3UTR_R**	GAAGTGACTGGGCAATGGTT	RT			
**Kap1_F**	CGGAAATGTGAGCGTGTTCTC	RT	1	73	Rowe et al., 2010
**Kap1_R**	CGGTAGCCAGCTGATGCAA	RT			
**MMERKV10C_gag_F**	ATGTGAGCTAGCTGTTAAAGAAGGAC	RT	11	72	Sharif et al., 2016
**MMERKV10C_gag_R**	CTCTCTGTTTCTGACATACTTTCCTGT	RT			
**Bglap3_F**	CTGACAAAGCCTTCATGTCC	RT	1	124	Ecco et al., 2016
**Bglap3_R**	TCAAGCTCACATAGCTCCC	RT			
**Cml2_F**	AGGTTTTACTGGATGTCATCGGA	RT	1	216	Choi et al., 2017
**Cml2_R**	CCCTGAGCCCTTTGGGAAC	RT			
**Serinc3_F**	TCTTCAAAATCGCTGCCATTATT	RT	1	88	Rowe et al., 2013
**Serinc3_R**	AGCAACAAACCAGACTTCAGTAAAAC	RT			
**Prnp_F**	CCTTCCTAGTGGTACCAGTCCAA	RT	1	70	Rowe et al., 2013
**Prnp_R**	AGCCAAGGTTCGCCATGA	RT			
**MMERKV10C_env_F**	TATCGCCTCAGGGTTAATGC	RT	30	101	Fasching et al., 2015
**MMERKV10C_env_R**	TGGATGCCACACAACTCATT	RT			
**Rgs20_IAPEY_F**	GTTCCTGCAAAACAGACTGC	RT	1	124	Ecco et al., 2016
**Rgs20_IAPEY_R**	ATTGCTCTGGTCAGCCATTC	RT			
**Rgs20_MMERVK_F**	ACTGGAGGTCCTTGTCCTATG	RT	1	103	Ecco et al., 2016
**Rgs20_MMERVK_R**	AGGTTCGATGTGCTCTTTCC	RT			
**MMERVK10Cint chr5_F**	GGGCAACTTTCCTCATGCAC	RT	1	245	this study
**MMERVK10Cint chr5_R**	AGGTGGTATAAGGCACTGTGG	RT			

**MMERVK10C_Zfp992__F**	TAATCTTTGGGCCAGGACTC	RT	1	126	Ecco et al., 2016
**MMERVK10C_Zfp992__R**	CCAAAGAAATGCCACACCTG	RT			
**Etn-ERVint (cml2)_F**	GATACACCCGGTTTGGATTG	RT	1	186	this study
**Etn-ERVint (cml2)_R**	TCCGTAGATGAGTGGGCTTT	RT			
**Rgs20 exon5-exon6-F**	GGATGGATCCACCATGTTTC	RT	3 isoforms	163	this study
**Rgs20 exon5-exon6-R**	CTGGATGGCCTGTGAAGAAC	RT			
**ZFP575_exon2_F**	CCTAATTCTCTGAGTCCCCAGTTC	RT	1	71	Rowe et al., 2013
**ZFP575_exon2_R**	AGTCTCAGCCTACTGATGCTGTGT	RT			
**Cml2 exon1-exon2/3_F**	GATCCAAATGTCCAGGATGG	RT	1	217	this study
**Cml2 exon1-exon2/3_R**	GTTTCCAAGATGCCTTTCCA	RT			
**Cyp2b23 LTR ETn-exon2_F**	CTGAACAGGGACATTGGGA	RT	1	290/390	Karimi et al., 2011
**Cyp2b23 LTR ETn-exon2_R**	CACTGGAAAAGATCACACCTA	RT			
**ETn_F**	GTGCTAACCCAACGCTGGTTC	RT	123	175	Karimi et al., 2011
**ETn_R**	CTCTGGCCTGAAACAACTCCTG	RT			
**MERVL_F**	ATCTCCTGGCACCTGGTATG	RT	641	50	Friedli et al., 2014
**MERVL_R**	AGAAGAAGGCATTTGCCAGA	RT			
**MTA_F**	ATGTTTTGGGGAGGACTGTG	RT	8	226	Karimi et al., 2011
**MTA_R**	AGCCCCAGTTAACCAGAAC	RT			
**Cntnap3_F**	CTTTGCCTCAGTTATCCTTCAGC	RT	1	161	this study
**Cntnap3_R**	GACCTCCATTCGTTCTCCAAG	RT			

* c = consensus primer; s = specific hit

References

1. Elsasser SJ, Noh KM, Diaz N, Allis CD, Banaszynski LA. Histone H3.3 is required for endogenous retroviral element silencing in embryonic stem cells. *Nature* **522**, 240-244 (2015).

2. Ding D*, et al.* The CUE1 domain of the SNF2-like chromatin remodeler SMARCAD1 mediates its association with KRAB-associated protein 1 (KAP1) and KAP1 target genes. *The Journal of biological chemistry* **293**, 2711-2724 (2018).

3. Karimi MM*, et al.* DNA methylation and SETDB1/H3K9me3 regulate predominantly distinct sets of genes, retroelements, and chimeric transcripts in mESCs. *Cell stem cell* **8**, 676-687 (2011).
