# Supplementary material for: SMARCAD1 ATPase activity is required to silence endogenous retroviruses in embryonic stem cells
Source: Nat Commun. 2019 Mar 22;10:1335. doi: 10.1038/s41467-019-09078-0 (PMC6430823; doi:10.1038/s41467-019-09078-0)
Supplement: Supplementary file 3 — Description of Additional Supplementary Files [file 41467_2019_9078_MOESM3_ESM.pdf]

Description of Additional Supplementary Files

Supplementary Movie 1

Dynamics of growth of ESCs expressing SMARCAD1.

Supplementary Movie 2

Dynamics of growth of ESCs lacking SMARCAD1.

E14 ESCs stably expressing an shRNA targeting the 3'UTR of SMARCAD1 (KD) and Control KD cells were analysed after their seeding in real time in 3 hour intervals for ~ 3 days. Shown are representative time lap movies acquired with IncuCyte ZOOM (Essen BioScience). Related to **Figure 1.**
