# Supplementary material for: SMARCAD1 ATPase activity is required to silence endogenous retroviruses in embryonic stem cells
Source: Nat Commun. 2019 Mar 22;10:1335. doi: 10.1038/s41467-019-09078-0 (PMC6430823; doi:10.1038/s41467-019-09078-0)
Supplement: Supplementary file 6 — Reporting Summary [file 41467_2019_9078_MOESM6_ESM.pdf]

nature research

Corresponding author(s): Jacqueline E Mermoud

Reporting Summary

Nature Research wishes to improve the reproducibility of the work that we publish. This form provides structure for consistency and transparency in reporting. For further information on Nature Research policies, see Authors & Referees and the Editorial Policy Checklist.

Statistical parameters

When statistical analyses are reported, confirm that the following items are present in the relevant location (e.g. figure legend, table legend, main text, or Methods section).

n/a	Confirmed	
☐	☒	The exact sample size (*n*) for each experimental group/condition, given as a discrete number and unit of measurement
☐	☒	An indication of whether measurements were taken from distinct samples or whether the same sample was measured repeatedly
☐	☒	The statistical test(s) used AND whether they are one- or two-sided<br>*Only common tests should be described solely by name; describe more complex techniques in the Methods section.*
☒	☐	A description of all covariates tested
☐	☒	A description of any assumptions or corrections, such as tests of normality and adjustment for multiple comparisons
☐	☒	A full description of the statistics including central tendency (e.g. means) or other basic estimates (e.g. regression coefficient) AND variation (e.g. standard deviation) or associated estimates of uncertainty (e.g. confidence intervals)
☐	☒	For null hypothesis testing, the test statistic (e.g. *F*, *t*, *r*) with confidence intervals, effect sizes, degrees of freedom and *P* value noted<br>*Give P values as exact values whenever suitable.*
☒	☐	For Bayesian analysis, information on the choice of priors and Markov chain Monte Carlo settings
☒	☐	For hierarchical and complex designs, identification of the appropriate level for tests and full reporting of outcomes
☒	☐	Estimates of effect sizes (e.g. Cohen's *d*, Pearson's *r*), indicating how they were calculated
☐	☒	Clearly defined error bars<br>*State explicitly what error bars represent (e.g. SD, SE, CI)*

Our web collection on statistics for biologists may be useful.

Software and code

Policy information about availability of computer code

Data collection	Deep sequencing was performed on the Illumina 1500 platform (Illumina).
Data analysis	Sequence alignment: Bowtie 1 version 0.12.7' and Bowtie 2

For manuscripts utilizing custom algorithms or software that are central to the research but not yet described in published literature, software must be made available to editors/reviewers upon request. We strongly encourage code deposition in a community repository (e.g. GitHub). See the Nature Research guidelines for submitting code & software for further information.

Data

Policy information about availability of data

All manuscripts must include a data availability statement. This statement should provide the following information, where applicable:

- Accession codes, unique identifiers, or web links for publicly available datasets
- A list of figures that have associated raw data
- A description of any restrictions on data availability

The data that support the findings of this study are available from the corresponding author upon reasonable request. Sequencing data has been deposited in the ArrayExpress repository under accessions E-MTAB-7011 (KAP1 ChIP-seq in E14 ESCs), E-MTAB-7012 (SMARCAD1 and H3K9me3 in PGK12.1 ESCs) and E-MTAB-7014

(FLAG-SMARCAD1 ChIP-seq in E14 ESCs). The source data underlying Figures 1a,1b,1g; Fig.5a; Fig.5b; Fig.6c,Fig.6g and Supplementary Figures 1f,2h,4f,6c,7c, 8b, 10b,c,f are provided in Supplementary Figure 11.

Field-specific reporting

Please select the best fit for your research. If you are not sure, read the appropriate sections before making your selection.

☒ Life sciences ☐ Behavioural & social sciences ☐ Ecological, evolutionary & environmental sciences

For a reference copy of the document with all sections, see nature.com/authors/policies/ReportingSummary-flat.pdf

Life sciences study design

All studies must disclose on these points even when the disclosure is negative.

Sample size	No statistical method was used to predetermine sample size. Sample size was based on traditional experimental approach in molecular and cell biology. In general, the sample sizes for qPCR, ChIP-qPCR were between 1-5 (repeated independently several times).
Data exclusions	In the comparison of the coverage of SMARCAD1 and KAP1 among ERV families the ChIP seq datasets were mismatched on sex, therefore repeats matching the sex chromosomes where filtered before comparison.
Replication	In Figure legends, number of replications was stated for each experiment.
Randomization	The experiments did not require sample randomization. Samples were handled the same way in all experiments.
Blinding	The investigators were not blinded during data collection or outcome assessment. This approach is considered standard for biochemical experiments performed in this study.

Reporting for specific materials, systems and methods

Materials & experimental systems

n/a	Involved in the study	
☒	☐	Unique biological materials
☐	☒	Antibodies
☐	☒	Eukaryotic cell lines
☒	☐	Palaeontology
☒	☐	Animals and other organisms
☒	☐	Human research participants

Methods

n/a	Involved in the study	
☐	☒	ChIP-seq
☐	☒	Flow cytometry
☒	☐	MRI-based neuroimaging

Antibodies

Antibodies used	All antibodies used in this study are listed in Supplementary Table 2 with their source and the method they were used in.
Validation	Antibodies suitable for specific purposes were purchased and the validation was performed by the vendors. In addition, key antibodies including Smarcad1, Kap1, Setdb1, were validated upon protein depletion by western blots, immunofluorescence and ChIP, depending on how the antibody was used in this study. The non-commercial anti-CUE1 SMARCAD1 Ab was validated in a publication from 2011 (Rowbotham et al., Mol Cell).

Eukaryotic cell lines

Policy information about cell lines

Cell line source(s)	The following cell lines were used in this study: E14 male mouse ESCs, J1 male mouse ESCs and PGK12.1 female mouse ESCs. All additional cell lines used are derivatives of one of these lines generated in the Mermoud lab.
Authentication	J1 mouse ES cells are from ATCC, E14 cells are described by Sharif et al., 2016. Cell Stem Cell, and PGK12.1 by Norris et al., 1994, Cell. Additional derivative cell lines generated in the Mermoud lab were validated by PCR.
Mycoplasma contamination	Mycoplasma contamination was assessed routinely using the MyoAlert Detection (Lonza) kit

Commonly misidentified lines
(See ICLAC register)

Name any commonly misidentified cell lines used in the study and provide a rationale for their use.

ChIP-seq

Data deposition

☒ Confirm that both raw and final processed data have been deposited in a public database such as GEO.

☐ Confirm that you have deposited or provided access to graph files (e.g. BED files) for the called peaks.

Data access links
May remain private before publication.

Array Express repository accessions E-MTAB-7011, E-MTAB-7012, E-MTAB-7014

Files in database submission

KAP1 ChIP-seq in E14 ESCs, SMARCAD1 and H3K9me3 in PGK12.1 ESCs, FLAG-SMARCAD1 ChIP-seq in E14 ESCs

Genome browser session
(e.g. UCSC)

Provide a link to an anonymized genome browser session for "Initial submission" and "Revised version" documents only, to enable peer review. Write "no longer applicable" for "Final submission" documents.

Methodology

Replicates

For ChIP-sequencing performed in E14 ESCs, ChIP experiments from three biological replicates were pooled before library preparation.

Sequencing depth

The ChIP-seq sequencing is described in detail in Supplementary Table 1 and in the methods, providing the total number of reads and the effective background reads.

Antibodies

SMARCAD1 PAB15737 Abnova, KAP1 ab22553 Abcam, IgG kCH-504 C15410206 Diagenode, FLAG F1804 Sigma, H3K9me3 ab8898 Abcam

Peak calling parameters

Peak calling was performed individually for each sample using antibody control or input as background. The MACS program v1.4.0rc2 72 was used for all samples, except for the histone modification mark H3K9me3. H3K9me3 ChIP peaks were called using SICER 1.1 73 with these parameters: windows size 200, gap size 200, fragment size 51, mappability percentage 0.78. For samples with corresponding antibody and input backgrounds only those peaks were kept that were called against both backgrounds.
Aligner: bowtie2 at Peak-calling and bowtie at Repeat-counting

Data quality

SMARCAD1 and KAP1 peaks were only retained if they had a minimum of 30 effective foreground reads, not more than 50 effective reads in either of their background(s), and showing at least a 2.5-fold increase in the normalized read counts (TPM) compared to their background(s). Only H3K9me3 peaks showing at least a three-fold increase in the normalized read counts compared to either of their backgrounds were kept.

Software

MACS program v1.4.0rc2, SICER 1.1, Bowtie 2.0.0-beta7, Integrative Genomics Viewer (IGV)

Flow Cytometry

Plots

Confirm that:

☐ The axis labels state the marker and fluorochrome used (e.g. CD4-FITC).

☐ The axis scales are clearly visible. Include numbers along axes only for bottom left plot of group (a 'group' is an analysis of identical markers).

☐ All plots are contour plots with outliers or pseudocolor plots.

☒ A numerical value for number of cells or percentage (with statistics) is provided.

Methodology

Sample preparation

ESCs resuspended in 200 µl of PBS were fixed by dropwise addition of 1.3 ml of ice-cold 70% ethanol. After fixation at 4 °C, cells were taken up in PI/RNase staining buffer (BD Pharmingen, 550825), incubated for 15 min at room temperature, and then analyzed.

Instrument

BD LSR II cytometer

Software

FlowJo version 10.2 software

Cell population abundance

The mESCs were sorted by the FlowJo software in order to exclude cell debris as well as doublets. This was determined by cell size and gating strategy. The Watson algorithm was used for determining final cell population abundance.

Gating strategy

The FlowJo software was used to plot the forward vs. the side scatter of the cell population. The cell population selected

Gating strategy	represented ~80% of the population and excluded cell debris (by size). Detected disproportions between cell size vs. cell signal were used for doublet discrimination and only single events were selected for cell cycle analysis. PerCP-A was set to linear and the Watson algorithm was used for final cell population abundance

☐ Tick this box to confirm that a figure exemplifying the gating strategy is provided in the Supplementary Information.
